# Cisplatin Induces a Mitochondrial-ROS Response That Contributes to Cytotoxicity Depending on Mitochondrial Redox Status and Bioenergetic Functions

**DOI:** 10.1371/journal.pone.0081162

**Published:** 2013-11-19

**Authors:** Rossella Marullo, Erica Werner, Natalya Degtyareva, Bryn Moore, Giuseppe Altavilla, Suresh S. Ramalingam, Paul W. Doetsch

**Affiliations:** 1 Department of Hematology and Medical Oncology, Emory University, Atlanta, Georgia, United States of America; 2 Department of Medical Oncology, University of Messina, Messina, Italy; 3 Department of Biochemistry, Emory University, Atlanta, Georgia, United States of America; 4 Geisiner Medical Center, Danville, Pennsylvania, United States of America; 5 Department of Radiation Oncology, Emory University, Atlanta, Georgia, United States of America; University of Pittsburgh, United States of America

## Abstract

Cisplatin is one of the most effective and widely used anticancer agents for the treatment of several types of tumors. The cytotoxic effect of cisplatin is thought to be mediated primarily by the generation of nuclear DNA adducts, which, if not repaired, cause cell death as a consequence of DNA replication and transcription blockage. However, the ability of cisplatin to induce nuclear DNA (nDNA) damage *per se* is not sufficient to explain its high degree of effectiveness nor the toxic effects exerted on normal, post-mitotic tissues. Oxidative damage has been observed *in vivo* following exposure to cisplatin in several tissues, suggesting a role for oxidative stress in the pathogenesis of cisplatin-induced dose-limiting toxicities. However, the mechanism of cisplatin-induced generation of ROS and their contribution to cisplatin cytotoxicity in normal and cancer cells is still poorly understood. By employing a panel of normal and cancer cell lines and the budding yeast *Saccharomyces cerevisiae* as model system, we show that exposure to cisplatin induces a mitochondrial-dependent ROS response that significantly enhances the cytotoxic effect caused by nDNA damage. ROS generation is independent of the amount of cisplatin-induced nDNA damage and occurs in mitochondria as a consequence of protein synthesis impairment. The contribution of cisplatin-induced mitochondrial dysfunction in determining its cytotoxic effect varies among cells and depends on mitochondrial redox status, mitochondrial DNA integrity and bioenergetic function. Thus, by manipulating these cellular parameters, we were able to enhance cisplatin cytotoxicity in cancer cells. This study provides a new mechanistic insight into cisplatin-induced cell killing and may lead to the design of novel therapeutic strategies to improve anticancer drug efficacy.

## Introduction

Cisplatin is one of the most effective and widely used drugs for the treatment of adult and pediatric cancers. Cisplatin is a highly reactive molecule that binds to RNA, DNA and proteins forming different types of adducts [1,2]; among these, adducts formed with nuclear DNA (nDNA) are considered the key lesions mediating the cytotoxic effect of cisplatin. Several intracellular DNA damage management pathways can either repair or tolerate these lesions. In particular, the nucleotide excision repair (NER) pathway plays a major role in removing cisplatin-nDNA adducts [3]; therefore, cells with compromised NER activity are extremely sensitive to cisplatin [4,5]. If not repaired, bulky nDNA adducts formed by cisplatin cause a block in nDNA replication and/or transcription, resulting in apoptosis [6–10]. This nDNA damage mediated mechanism of cell killing explains the high toxicity of cisplatin in dividing cells. However, cisplatin exposure also results in severe damage to post-mitotic tissues. A major limitation in cisplatin therapeutic use is the development of toxicities that impair the function of cells in kidney, ear and sensory nerves [11–13]. Toxicity of cisplatin does not entirely depend on the amount of drug accumulation in normal tissues [14], suggesting that blockage of nDNA transcription may not be the only mechanism determining the toxic effect of cisplatin in non-replicating cells. Thus, cisplatin-induced generation of nDNA damage *per se* is not sufficient to explain its high degree of effectiveness as an anticancer agent as well as the tissue specificity of its cytotoxic effects on normal, post-mitotic tissues. 

Cisplatin accumulates in mitochondria and forms adducts with mitochondrial DNA (mtDNA) and proteins [15,16]. Several reports have compared the vulnerability of nDNA and mtDNA to cisplatin-induced DNA damage with notably inconsistent results (recently reviewed in 17); therefore, mtDNA susceptibility to cisplatin damage may depend on specific cellular/tissue characteristics. Depletion of mitochondrial DNA has been observed to modulate cellular sensitivity to cisplatin with controversial results [16,18–20]. Mitochondria, whose main function is to produce energy by oxidative phosphorylation, are also one of the most important endogenous sources of reactive oxygen species (ROS). Cisplatin exposure results in intracellular ROS increase in normal cells [21–23] and treatment with antioxidants ameliorates the toxic effects of cisplatin on several organs [24–27], suggesting an involvement of oxidative stress in the pathogenesis of cisplatin-induced dose-limiting toxicities. However, little is known about the mechanism of cisplatin-induced generation of ROS and how such an oxidative stress response affects normal and cancer cell sensitivity to the drug.

The goal of the present study was to elucidate the role of mitochondria in the oxidative stress response to cisplatin exposure and to determine the contribution of ROS generation in determining cisplatin cytotoxicity in normal and cancer cells. We also sought to identify key modulators of cellular sensitivity to this ROS-mediated component of cisplatin cytotoxicity.

By revealing new mechanistic insights of cisplatin-induced cytotoxicity our results are valuable for the design of therapeutic strategies to improve cisplatin anticancer efficacy in tumor cells as well as to prevent and/or limit the onset of cisplatin dose-limiting toxicities. 

## Materials and Methods

### Cell lines

 DU145 and its derivative DU145ρ^°^ prostate cancer cell lines were donated by John Petros, MD, Emory University [28]. DU145 cells were cultured in 10% FBS RPMI1640; ρ^0^ status of DU145ρ^°^ cells was confirmed as described in Supporting Information (Figure S1) and cells were maintained in 10% RPMI1640 with glucose (200 mg/mL), sodium pyruvate (11 mg/mL) and uridine (5 mg/mL). A549 cell line was purchased from ATCC and maintained in DMEM medium with 10% FBS. WT and TFAM^+/-^ MEFs cell lines were provided by David Martin, PhD, (Emory University) [29] and cultured as described [29]. All the cell lines were grown at 37°C in 5% CO_2_ humidified incubators. 

### Assessment of ROS levels in mammalian cells

Mammalian cells were plated on a 6 well plate the day before the experiment. Media was then replaced with fresh media containing cisplatin, carboplatin or chloramphenicol. Following exposure to the drug, ROS levels were assessed by incubating cells with either H_2_DCFDA (10 μM; Sigma-Aldrich) or MitoSox (5 μM; Molecular Probes) for 30 min at 37°C. When used as positive control, hydrogen peroxide (2 μM) was added to the labeled cells 15 minutes before fluorescence measurement.

Simultaneous detection of mitochondrial ROS and apoptosis was carried out as previously described [30] and shown in Supporting Information (Figure S2), using Alexa Fluor 488/Annexin V (Molecular Probes) as a marker of apoptosis. When used as positive control, Antimycin A (2 μg/mL; Sigma-Aldrich) was added at the same time of probe addition. 

Following incubation with fluorescent probe(s) cells were washed twice resuspended in PBS and assessed for fluorescence intensity by employing a BD LSR II flow cytometer (BD Biosciences). Data were analyzed using FlowJo Software.

### Survival analysis in mammalian cells

Mammalian cells were plated on a 96 well plate the day before the experiment. Media was replaced with fresh media containing a dose range of cisplatin (Sigma-Aldrich) or carboplatin (Sigma-Aldrich). For co-treatment experiments, a fixed dose of NAC (1 mM; Sigma-Aldrich) or DCA (1 mM; Sigma-Aldrich) was added at the same time of cisplatin or carboplatin treatment. Following 72 h of exposure, media was removed and cells were fixed with methanol for 10 minutes. Cells were subsequently stained with 0.1% crystal violet for 15 minutes, washed and solubilized with 100 μL of 2% sodium deoxycholate. Absorbance was measured using a SpectraMax M5 Plate Reader. 

### Yeast strains

In this study we used the yeast strains: SJR751 (*MATa ade2-101*_*oc*_ *his3Δ200 ura3ΔNco lys2ΔBgl leu2-R*) and SJR868 (*MATa ade2-101*_*oc*_ *his3Δ200 ura3DNco lys2ΔBgl leu2-R rad1Δ::hisG*) [31]. Yeast cells were grown on yeast extract-peptone-dextrose (YPD) medium. WTρ^°^ and NER^-^ρ^0^ strains were generated by incubating cells in ethidium bromide as previously described [32]. ρ^0^ status was verified by inability of growth on yeast extract-peptone-glycerol (YPG) medium. Lack of mitochondrial DNA was further confirmed by 4',6-diamidino-2-phenylindole (DAPI, Sigma-Aldrich) and MitoTracker Red staining (Invitrogen) (data not shown).

### Assessment of ROS levels and viability in yeast cells

Yeast cells were grown in YPD at 30°C overnight. Cells were then counted, adjusted to 2×10^7^ cells/mL and exposed to 100 μM of cisplatin in the dark for 1.5 hours at 30°C. ROS levels were assessed using the fluorescent probe dihydroethidium (50 μg/mL; Molecular Probes) as previously described [33].

For survival measurement, cells were plated in duplicate on YPD plates at a density of 100–200 colonies per plate and incubated at 30°C for 48 h. 

### Electron transport chain protein expression analysis

Cells were plated on a 100 mm dish the day before the experiment. Cells were then exposed to cisplatin (12 μM), carboplatin (50 μM) or chloramphenicol (100 μg/mL) for 24 h and a mitochondrial enriched cell lysate was prepared as previously described [34].

For SDS-PAGE and Western Blot analysis, 7.5 µg of protein were boiled with 6× SDS-PAGE loading buffer and samples were separated on precast NU-PAGE 10% Bis-Tris minigels (Novex). Western Blot analysis was performed with primary anti-SDHA antibody (0.1 μg/mL; MitoSciences) or with primary anti-MT-CO1 antibody (1 μg/mL; MitoSciences) diluted in blocking solution (5% milk in TBST). HRP-conjugated anti-mouse (1:5000; Promega) was employed as secondary antibody. Chemiluminescence was used to detect immunoreactive proteins, and protein abundance was quantified based on band intensities using ImageQuant software.

### RNA extraction, reverse transcription and Real-Time PCR analysis

Cells were plated on a 100 mm dish the day before the experiment. Cells were then exposed to cisplatin (12 μM), carboplatin (50 μM) or ethidium bromide (100 μg/mL) for 24 h and total RNA was extracted with TRIzol Reagent (Life Technologies) following the manufacturer’s protocol and quantified with NanoDrop 2000 spectrophotometer (Thermo Scientific). 1 μg of isolated RNA was treated with DNAase I (Life Technologies) for 20 minutes at 37°C to remove genomic DNA contamination and converted to cDNA by using AccuScript High Fidelity 1^st^ Strand cDNA Synthesis (Agilent Technologies) according to the manufacturer’s protocol. cDNA amplification was carried out by real-time PCR using the StepOnePlus™ Real-Time PCR System (Applied Biosystems) with the following cycling conditions: 10 minutes at 95°C, 40 cycles of 15 seconds at 95°C, 30 seconds at 57°C and 30 seconds at 72°C. A dissociation curve analysis was performed for each sample at the end of each profile to verify PCR specificity. Mock reverse transcription and no template samples were used as negative controls. The PCR reaction contained 12.5 μL of QuantiTect SYBR Green PCR MasterMix (Qiagen), 1 μL of target-specific primers for MT-CO1 (10 μM stock) and SDHA (10 μM stock) and 1.2 μL for RPLP0 (10 μM stock), 1 μL of cDNA and water up to 25 μL of volume. Primers for MT-CO1 and SDHA were purchased from Qiagen (RT^2^ qPCR Primer Assay, cat #PPH60272E and #PPH20936F, respectively); primer sequences for RPLP0 gene were the following: 5’ GGGCGACCTGGAAGTCCAACT 3’ (forward), 5’ CCCATCAGCACCACAGCCTTC 3’ (reverse). 

 The average mRNA fold change following exposure to cisplatin, carboplatin and ethidium bromide was calculated by the ΔΔCt method using RPLP0 as internal control and non treated samples as calibrator [35]. Five biological replicates were analyzed for each treatment; samples were run in triplicates and were averaged prior to analysis. 

### Generation of cells expressing mitochondrial-targeted catalase

 A plasmid containing a mitochondrial-targeted catalase was a gift from Dr. Andrew Melendez (Albany Medical College) [36]. A549 cells were transfected with 4 μg of DNA using Lipofectamine 2000 (Life Technologies) according to the manufacturer’s instructions. 48 h following transfection, cells were passaged into a 75 cm^2^ flask and selective media (Zeocin, 4 μg/µL, Invitrogen) was added. Expression of mitochondrial-targeted catalase (mCat) in transfected cells was evaluated as described in Supporting Information (Figure S3).

### Data representation and statistical analysis

 Data represent the mean of three independent experiments, each performed in triplicate and error bars represent standard deviation (unless otherwise indicated). Statistical analyses were performed by using GraphPad Prism 5.0. One-way ANOVA and two-way ANOVA followed by Bonferroni post-test for multiple comparisons were used to analyze ROS levels and mRNA expression following exposure to platinum-drugs. Cell survival was compared by two-tailed Student t-test with Bonferroni correction for multiple comparisons. 

## Results

### 1. Cisplatin exposure induces a mitochondria-dependent increase in reactive oxygen species levels in cancer cells

 To gain insight into the source and the potential mechanism of cisplatin-induced generation of ROS in human cells, we analyzed the temporal nature of this process. We used the non-small cell lung cancer cell line A549, as cisplatin-based chemotherapy is a standard of care for this type of tumor [37]. In order to determine the role of mitochondria in cisplatin-induced ROS generation we utilized the prostate cancer cell lines DU145 and its isogenic ρ^0^ derivative (DU145ρ^°^; ρ^0^ status was validated as described in Figure S1). 

**Figure 1:**
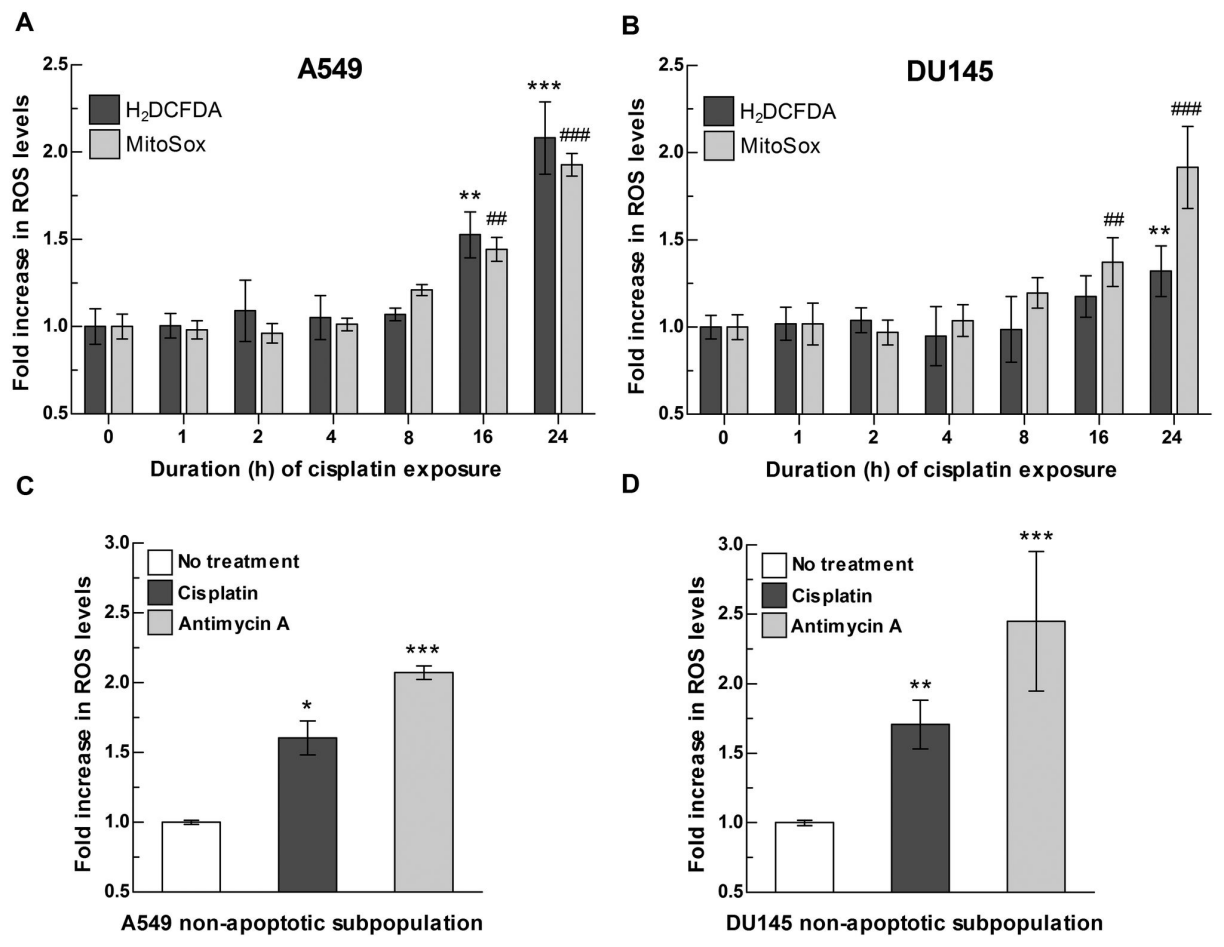
Cisplatin exposure induces an increase in total intracellular and mitochondrial ROS in non-apoptotic cancer cells. (**A**-**B**) Temporal analysis of ROS levels following cisplatin exposure in (**A**) A549 and (**B**) DU145 cells. A549 and DU145 cells were exposed to cisplatin at an IC50 dose (12 μM and 20 μM, respectively) and ROS levels were measured at the indicated time points by incubating with H_2_DCFDA or MitoSox fluorescent probes. ROS levels in treated vs. non treated cells at each time point were analyzed independently for each probe by two-way ANOVA. For total intracellular ROS levels (H_2_DCFDA): treatment x time point interaction p<0.05 for DU145 cells and p<0.001 for A549 cells; Bonferroni post-test for multiple comparison: ** p<0.01, *** p<0.001. For mitochondrial ROS (MitoSox): treatment x time interaction p<0.001 for DU145 and A549 cells; Bonferroni post-test for multiple comparison: ## p<0.01, ### p<0.001. (**C**-**D**) ROS levels following cisplatin exposure in non-apoptotic (**C**) A549 and (**D**) DU145 cells. A549 and DU145 cells were exposed to cisplatin at an IC50 dose and ROS levels measured in Annexin V-negative subpopulation as described in Materials and Methods and in Figure S2. Antimycin A was used as positive control for mitochondrial ROS generation. ROS levels in treated vs. non treated cells were analyzed by one-way ANOVA (p<0.001 A549 and DU145 cells; Bonferroni post-test for multiple comparison: *p<0.05, **p<0.01, *** p<0.0001). Data are presented as fold increase over no treatment. Bars represent the mean of n=3-6 independent biological replicates +/- SD.

A549 and DU145 cells were continuously exposed to cisplatin at an IC50 dose for 24 h and intracellular and mitochondrial levels of ROS were measured throughout the exposure period. By using the 2',7'-dichlorodihydrofluorescein diacetate probe (H_2_DCFDA) that reacts with multiple ROS species in the cells, we observed a significant increase in intracellular ROS levels at 16 h following initial cisplatin exposure and continuously increasing up to 24 h (Figure 1A-B). A parallel increase in mitochondrial superoxide was observed by using the mitochondrial-specific probe MitoSox. Such a cisplatin-induced increase in mitochondrial ROS is not only due to apoptosis, as we were able to detect a significant increase in mitochondrial ROS levels in the non-apoptotic subgroup of cells exposed to the drug (Figure 1C-D and Figure S2). These data suggest that mitochondria are the source of cisplatin-induced ROS response in cancer cells. 

**Figure 2:**
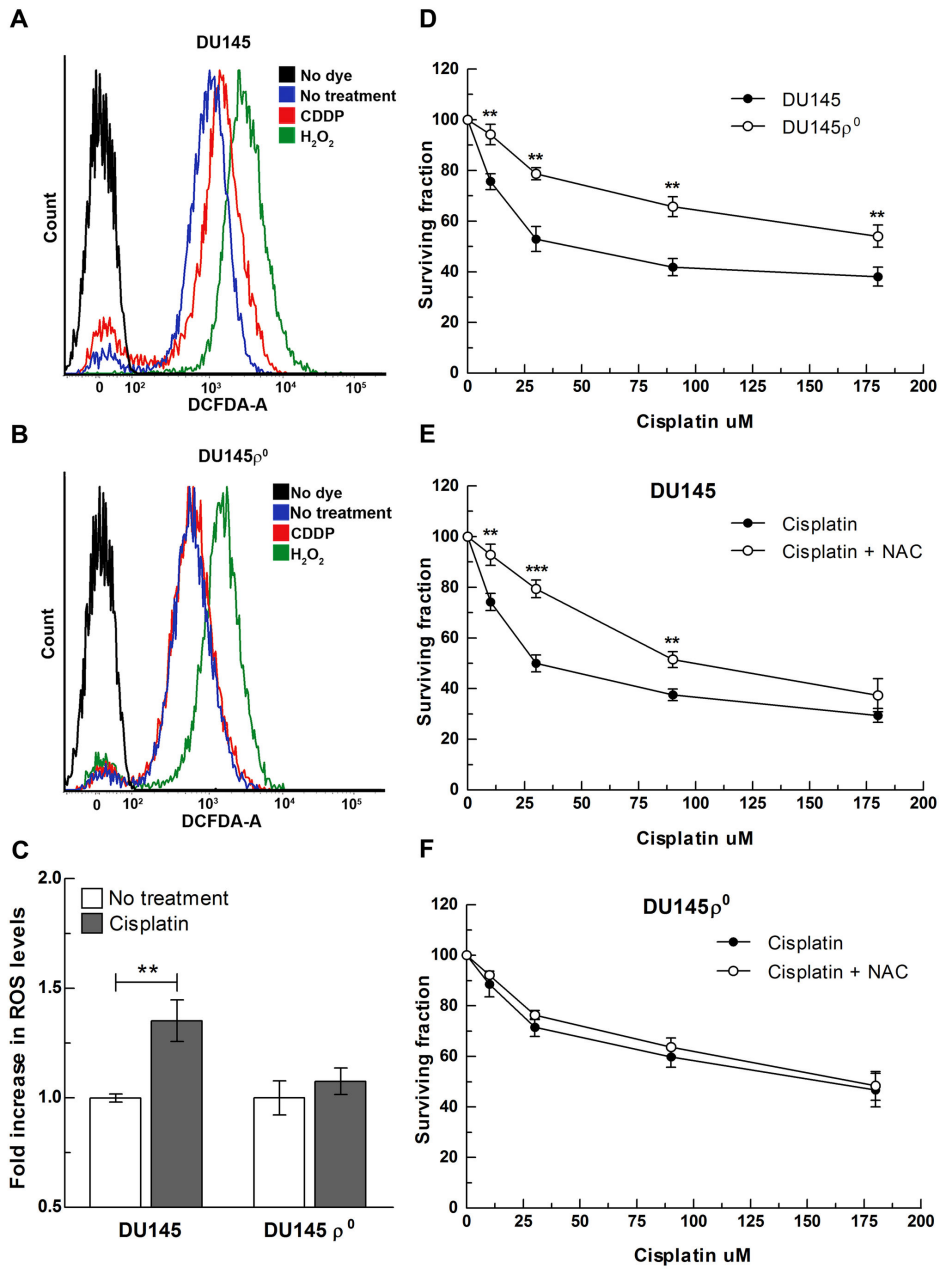
(A-C) Mitochondria are the source of cisplatin-induced ROS in cancer cells. Representative flow cytometry curves of total intracellular ROS levels (H_2_DCFDA) in (**A**) DU145 and (**B**) isogenic DU145ρ^°^ cells following 24 h of exposure to cisplatin at an IC50 dose (20 μM). H_2_O_2_ was used as positive control. (**C**) Quantitative representation of previous experiment. Data are presented as fold increase over no treatment. Bars represent the mean of n=3 independent biological replicates +/- SD. ROS levels in treated vs. non treated cells in DU145 and DU145ρ^°^ genotypes were analyzed by two-way ANOVA (treatment x genotype interaction p<0.05; Bonferroni post-test for multiple comparison: ** p<0.01). (**D**-**F**) **Mitochondrial ROS contribute to the cell killing effect of cisplatin**. (**D**) Survival of DU145 and isogenic DU145ρ^°^ after exposure to a dose range of cisplatin. (**E**) DU145 and (**F**) DU145ρ^°^ cell survival after exposure to a dose range of cisplatin with or without 1 mM of NAC. Data represent mean of n=3 independent experiments +/- SD. **p<0.005, ***p<0.0005.

To support this hypothesis, ROS levels were measured following cisplatin exposure in DU145ρ^°^ cells. DU145ρ^°^ cells lack mitochondrial DNA and, as a consequence, are respiratory incompetent. Although mitochondria are a major endogenous source of ROS, DU145ρ^°^ cells display only ~20% lower endogenous levels of ROS compared to the parental cells (Figure S4). Since ROS are known to mediate important physiological processes, other sources of ROS may have been activated and/or expressed in ρ^0^ cells as a compensatory mechanism. When intracellular ROS levels were measured 24 h following cisplatin exposure, no change was observed in DU145ρ^°^ cells, indicating that mitochondria are indeed the source of ROS in cancer cells (Figure 2A-C).

### 2. Cisplatin-induced mitochondrial ROS response enhances the cytotoxic effect caused by nuclear DNA damage

 In order to evaluate the biological effect of cisplatin-induced generation of ROS in cancer cells, we compared the cytotoxicity profiles of DU145 and DU145ρ^°^ cells following exposure to cisplatin. DU145ρ^°^ cells showed reduced sensitivity toward cisplatin compared to the parental cells (Figure 2D). This differential sensitivity is not due to impairment in the apoptotic response as ρ^0^ cells are still susceptible to apoptosis following exposure to staurosporine, with a magnitude of response comparable to the parental DU145 cell line (Figure S5). To verify that mitochondria-generated ROS are contributing to cisplatin-induced cell killing, we evaluated the effect of the antioxidant N-acetyl-cysteine (NAC) on cell survival following cisplatin exposure. The exposure to NAC reduced DU145 sensitivity to cisplatin, without affecting the sensitivity of DU145ρ^°^ cells (Figure 2E-F). Collectively, these results demonstrate that the mitochondrial ROS response is necessary for full expression of cisplatin cytotoxicity. 

### 3. Exposure to cisplatin induces ROS generation by a mechanism independent of nDNA damage signaling in *Saccharomyces cerevisiae*

**Figure 3:**
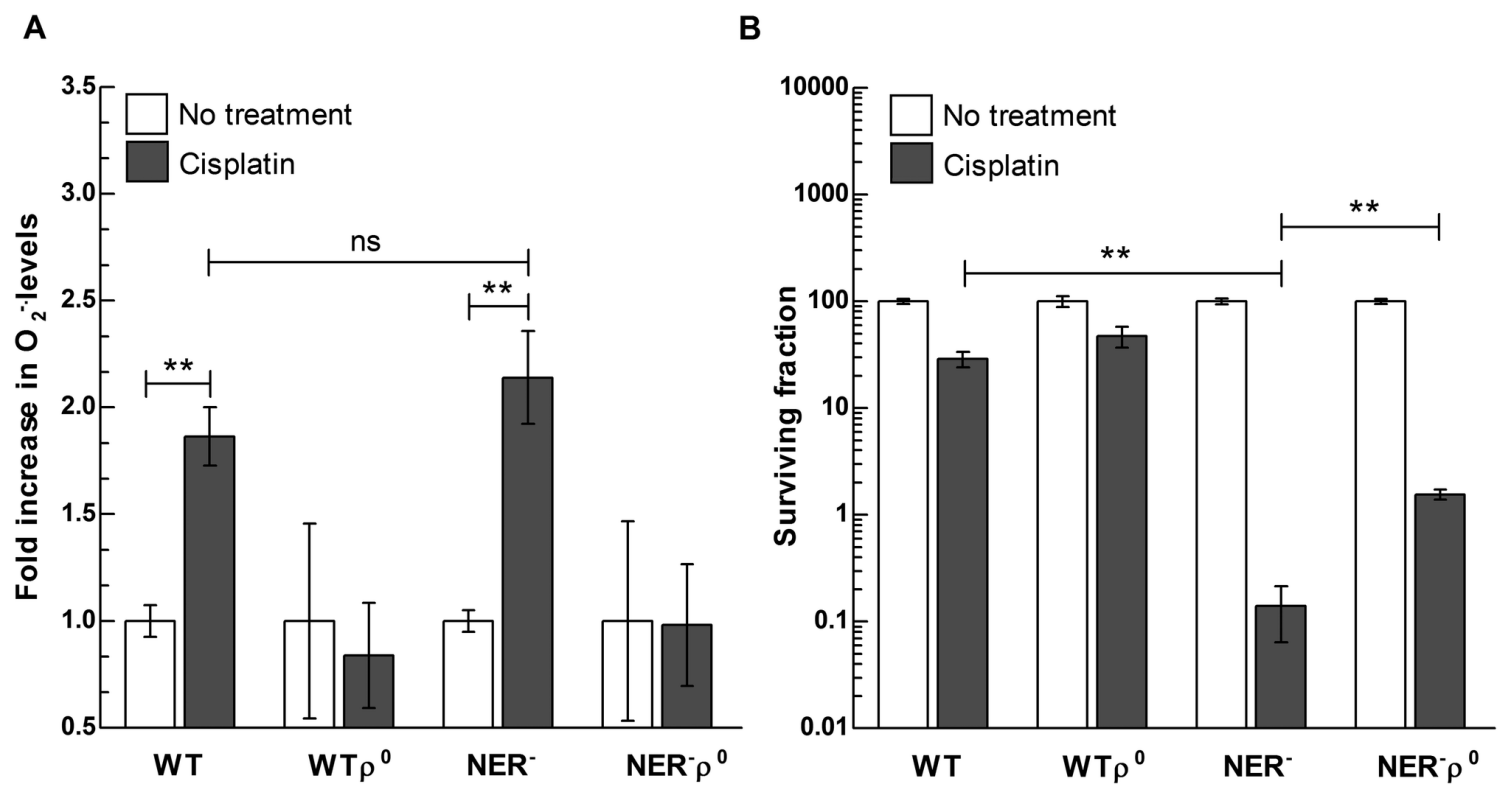
Cisplatin-induced mitochondria-dependent increases in ROS levels occur by a mechanism independent of nDNA damage signaling. WT and NER^-^ cells and their isogenic WTρ^°^ and NER^-^ρ^0^ genotypes were exposed to cisplatin (100 μM) for 2 h and (**A**) intracellular ROS levels were measured by incubating with dihydroethidium fluorescence probe. Bars represent the mean of n=3-6 independent biological replicates +/- SD. ROS levels in treated vs. non treated cells in WT, WTρ^°^, NER^-^ and NER^-^ρ^0^ strains were analyzed by two-way ANOVA (treatment x genotype interaction p<0.05; Bonferroni post-test for multiple comparison:** p<0.01). (**B**) Viability of WT and NER^-^ cells and their isogenic WTρ^°^ and NER^-^ρ^0^ strains after exposure to cisplatin (100 μM). Bars represent the mean of n=3-6 independent biological replicates +/- SD. Data were analyzed with Student t-test with Bonferroni correction for multiple comparisons; ns: not significant, **p<0.001.

Our findings indicate that mitochondria are the source of cisplatin-induced ROS generation. Damage to nDNA induces a ROS-mediated stress response in yeast and mammalian cells [33,38]. Therefore, cisplatin-induced generation of ROS in mitochondria may occur either as a signaling response to nuclear DNA damage or as a direct effect of cisplatin on mitochondria. To distinguish between these two possibilities, we employed the budding yeast *Saccharomyces cerevisiae*, a genetically tractable organism that allowed generation of isogenic strains lacking mitochondrial DNA (ρ^0^) within nDNA damage repair proficient (WT) or deficient (NER^-^) backgrounds. We reasoned that if the increase of mitochondrial ROS occurs as a response to cisplatin-induced nDNA damage, NER^-^ cells should display a significantly higher increase in ROS levels following exposure to cisplatin. By employing a model organism we also sought to evaluate whether the observed mitochondrial-ROS generation induced by cisplatin is a general eukaryotic cellular response or, instead, is species/cell type-specific. Following cisplatin exposure, no increase in ROS levels was observed in either WTρ^°^ or NER^-^ρ^0^ strains (Figure 3A), indicating that mitochondria are the source of cisplatin-induced ROS generation in yeast, as in human cells. A similar magnitude of increase in cellular ROS levels was observed in WT and NER^-^ strains, regardless of their DNA repair background (Figure 3A); therefore, the ROS response is independent of the ability of cells to repair cisplatin-induced nDNA damage. These results indicate that the observed generation of ROS in mitochondria is likely to be a general eukaryotic cellular response to cisplatin exposure that is not correlated with the level of nDNA damage caused by the drug.

By using this approach we were also able to dissect the respective contributions of nDNA damage and mitochondrial-ROS generation in determining cisplatin cytotoxicity. As expected, NER^-^ cells are extremely sensitive to cisplatin, due to their inability in repair cisplatin-induced nDNA damage. Yeast cells lacking functional mitochondria display lower sensitivity to cisplatin compared to the isogenic respiratory proficient strains (Figure 3B), similar to what we observed in human cancer cells. However, the difference in viability among respiration-competent and incompetent cells was minimal in the repair proficient background, suggesting that the ROS increase may be necessary but not sufficient to induce cell death in yeast. 

### 4. Exposure to cisplatin impairs the synthesis of electron transport chain proteins encoded by mitochondrial DNA

Our findings indicate that cisplatin-induced increase in ROS levels does not occur in response to nDNA damage signaling, but instead may be caused by direct damage to mitochondria. Cisplatin binds mitochondrial DNA (mtDNA) as efficiently as nDNA, but it is unlikely to be removed, as mitochondria lack NER [15]. The persistence of unrepaired cisplatin-induced mtDNA adducts could interfere with mtDNA transcription, resulting in a reduction of protein synthesis. Reduced expression of mtDNA encoded components of the electron transport chain (ETC) would impair respiration and subsequently lead to ROS generation. The time course of the increase in ROS (Figure 1A-B) is consistent with such a mechanism. 

**Figure 4:**
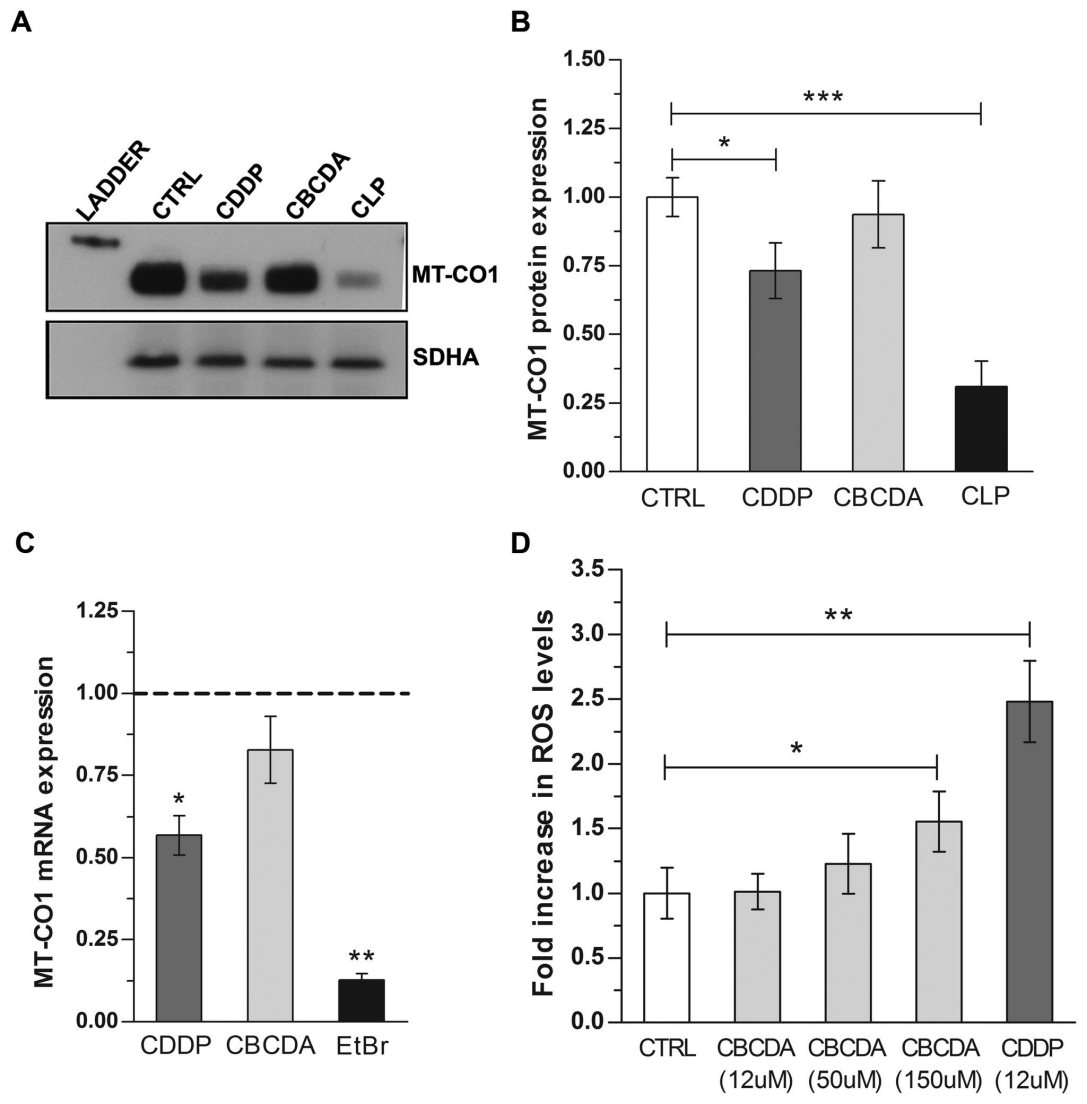
(A-B) Cisplatin exposure reduces the expression of mitochondrial-DNA encoded proteins. (**A**) Representative Western Blot of mitochondrial-encoded cytochrome c oxidase subunit 1 (MT-CO1) and succinate dehydrogenase subunit A (SDHA) expression in A549 cells exposed to cisplatin (CDDP) or carboplatin (CBCDA) at a IC50 dose (12 μM and 50 μM, respectively) for 24 h. Chloramphenicol (CLP) at 100 μg/mL was used as positive control. (**B**) Quantitative analysis of n=3 independent biological replicates. MT-CO1 expression was normalized over SDHA expression. Data are presented as fold change over control (no treatment). (**C**-**D**) **Carboplatin is less efficient than cisplatin in impairing mtDNA transcription and generating ROS in cancer cells**. (**C**) MT-CO1 mRNA levels following exposure to cisplatin and carboplatin. A549 cells were exposed to cisplatin and carboplatin at an IC50 dose (12 μM and 50 μM, respectively) and mRNA levels analyzed by qRT-PCR as described in Materials and Methods. Bar represent mean of n=5 experiments +/- SEM. Data are presented as fold change compared to control (no treatment, black dotted line). MT-CO1 mRNA expression levels in treated vs. non treated cells were analyzed by one-way ANOVA (p<0.005; Bonferroni post-test for multiple comparison:* p<0.05; **p<0.005 (**D**) ROS levels in A549 following exposure to cisplatin and carboplatin. A549 cells were exposed to cisplatin (CDDP) at an IC50 dose (12 µM) or a range of carboplatin (CBCDA) doses and total intracellular ROS levels were measured after 24 h by incubating with H_2_DCFDA. ROS levels in treated vs. non treated cells were analyzed by one-way ANOVA (p<0.005; Bonferroni post-test for multiple comparison: *p<0.05, **p<0.01). Data are presented as fold increase over control (no treatment). Bars represent the mean of n=3 independent biological replicates +/- SD.

To investigate this possibility, we analyzed the effect of cisplatin exposure on mitochondrial protein steady-state levels. We exposed A549 cells to cisplatin for the duration of time at which we observed the highest level of ROS (24 h), and measured the expression levels of ETC proteins encoded by mtDNA genes. As a negative control, we analyzed the expression of ETC subunits encoded by nDNA genes. As a positive control, we assessed the cellular expression of ETC proteins following exposure to chloramphenicol, an antibiotic that inhibits mitochondrial protein translation [39]. Following exposure to cisplatin at an IC50 dose, we detected a reduction in the expression of the mitochondrial-encoded cytochrome c oxidase subunit 1 (MT-CO1), while no changes were observed in the expression of the nDNA-encoded protein succinate dehydrogenase subunit A (SDHA) (Figure 4A-B). A similar result was observed in cells when mitochondrial protein synthesis was inhibited with chloramphenicol and consistent with this disruption, exposure to chloramphenicol increased mitochondrial ROS levels over a time course similar to cisplatin (Figure S6). These data indicate that cisplatin causes a reduction in mtDNA-encoded protein synthesis that is likely a consequence of mtDNA transcription blockage caused by mtDNA adducts. To further investigate this mechanism we analyzed the mRNA levels of MT-CO1 and SDHA following exposure to cisplatin. Ethidium bromide, a DNA intercalating agent that inhibits mtDNA transcription and replication, was used as positive control. A significant reduction in MT-CO1 mRNA levels was observed in cells treated with cisplatin [0.55 (0.47-0.64)] and ethidium bromide [0.12 (0.09-0.16)] compared to non treated control [1 (0.8-0.14)] (Figure 4C). No significant change was observed in SDHA mRNA levels following cisplatin exposure (Figure S7).

To confirm the essential role of mitochondrial protein synthesis impairment in enhancing cisplatin cytotoxicity, we evaluated the effects of another platinum-based drug, carboplatin, which forms similar types of nDNA lesions, but is substantially less toxic than cisplatin [40]. Consistent with previous reports, we observed that the IC50 of carboplatin is about 4 fold higher than cisplatin in A549 cells (IC50: 12 μM and 50 μM, respectively). Treatments with an IC50 dose of carboplatin did not significantly reduce the expression of mtDNA encoded protein MT-CO1 (Figure 4A-B) as well as MT-CO1 mRNA levels (Figure 4C). Accordingly, carboplatin exposure did not increase ROS levels at this dose (Figure 4D). These results indicate that cisplatin-induced generation of ROS occurs in mitochondria as a consequence of mtDNA transcription block, which leads to a subsequent reduction in protein synthesis and impairment in ETC function.

### 5. Mitochondrial redox status, DNA integrity and metabolic activity influence the cellular response to cisplatin-induced mitochondrial damage

**Figure 5:**
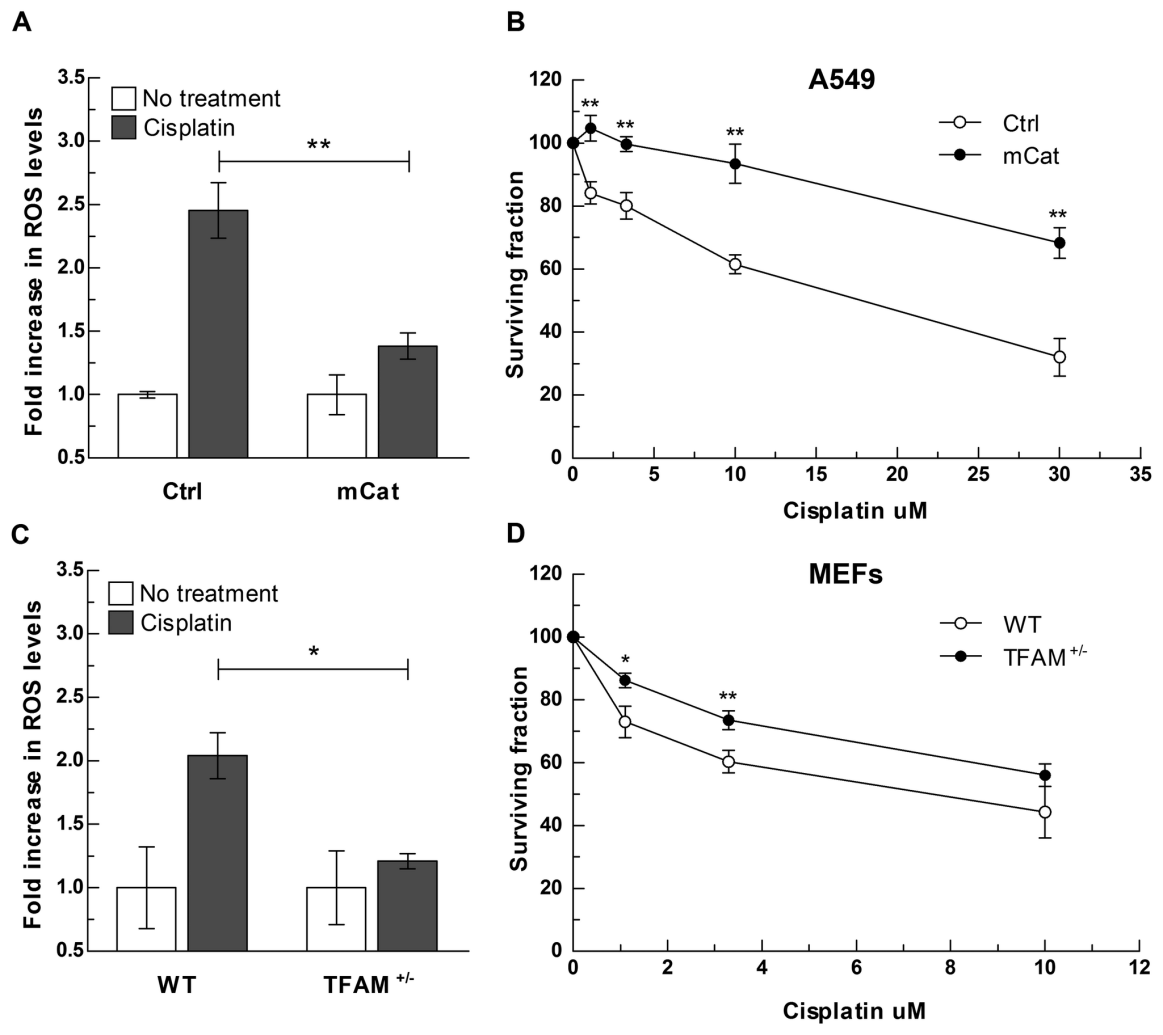
(A-B) Cells with increased expression of catalase in mitochondria are less sensitive to cisplatin. (**A**) mCat and isogenic Ctrl cells were exposed to cisplatin (12 μM) for 24 h and ROS levels measured by incubating with H_2_DCFDA. Data are presented as fold increase over no treatment. Bars represent the mean of n=3 independent biological replicates +/- SD. ROS levels in treated vs. non treated cells in Ctrl and mCat genotypes were analyzed by two-way ANOVA (treatment x genotype interaction p<0.001; Bonferroni post-test for multiple comparison: ** p<0.001). (**B**) Survival of mCat cells and Ctrl cells following 72 h of exposure to a dose range of cisplatin. Data represent mean of n=3 independent experiments +/- SD; ** p<0.005. (**C**-**D**) **Cells with dysfunctional mitochondria are less sensitive to cisplatin**. (**C**) WT and TFAM^+/-^ MEFs were exposed to cisplatin (10 μM) for 24 h and ROS levels measured by Amplex Red as described in Materials and Methods S1. Data are presented as fold increase over no treatment. Bars represent the mean of n=3 independent biological replicates +/- SD. ROS levels in treated vs. non treated cells in WT and TFAM^+/-^ genotypes were analyzed by two-way ANOVA (treatment x genotype interaction p<0.05; Bonferroni post-test for multiple comparison: * p<0.05). ROS levels in exposed to cisplatin at an IC50 dose (10 μM) for 24 h. ROS levels were measured (**D**) Survival of WT and TFAM^+/-^ MEFs exposed to a dose range of cisplatin. *p<0.05, p<0.005.

Our data indicate that cisplatin-induced mitochondrial ROS generation significantly contributes to its cytotoxicity. Therefore, alteration of mitochondrial redox status by modulation of ROS scavenging capacity may influence cellular sensitivity to cisplatin. In order to determine whether a targeted increase in mitochondrial ROS scavenging capacity affects cisplatin cytotoxicity, we transfected A549 cells with a plasmid containing a mitochondrial-targeted catalase gene (Figure S3A-B). Cells expressing catalase in mitochondria (mCat) had lower endogenous ROS levels compared to non-transfected cells (Ctrl; Figure S3C) and lower intracellular ROS levels in response to cisplatin (Figure 5A). mCat cells displayed reduced sensitivity to cisplatin (Figure 5B), indicating that efficient removal of mitochondrial ROS constitutes a potential mechanism of resistance to the cytotoxic effect of cisplatin. Similarly, co-treatment with the mitochondrial ROS scavenger Mitotempo reduced cisplatin-induced apoptosis in A549 cells (Figure S8).

 Our results indicate that cells lacking functional mitochondria are more resistant to cell killing by cisplatin (Figure 2D). To determine whether mitochondrial DNA integrity affects responses to cisplatin involving specific cellular operational pathways, we employed mouse embryonic fibroblasts (MEFs) heterozygous for the mitochondrial transcription factor A gene (TFAM^+/-^). TFAM^+/-^ are a well established model for cells with mitochondrial genome instability and altered mitochondrial function. TFAM is a mitochondrial transcription factor that regulates mtDNA transcription and replication [41]. TFAM^+/-^ MEFs express lower amount of TFAM protein, harbor about 50% less mtDNA and have increased oxidative mtDNA damage [29]. TFAM deficiency results in reduced respiratory chain function and increased expression of glycolytic enzymes [42]. Cisplatin treatment induced a lower ROS response and toxicity in TFAM^+/-^ cells compared to the isogenic WT cells (Figure 5C-D), indicating that mitochondrial dysfunction(s) may confer resistance to cisplatin as a consequence of the absence/reduction of the mitochondrial ROS response.

**Figure 6:**
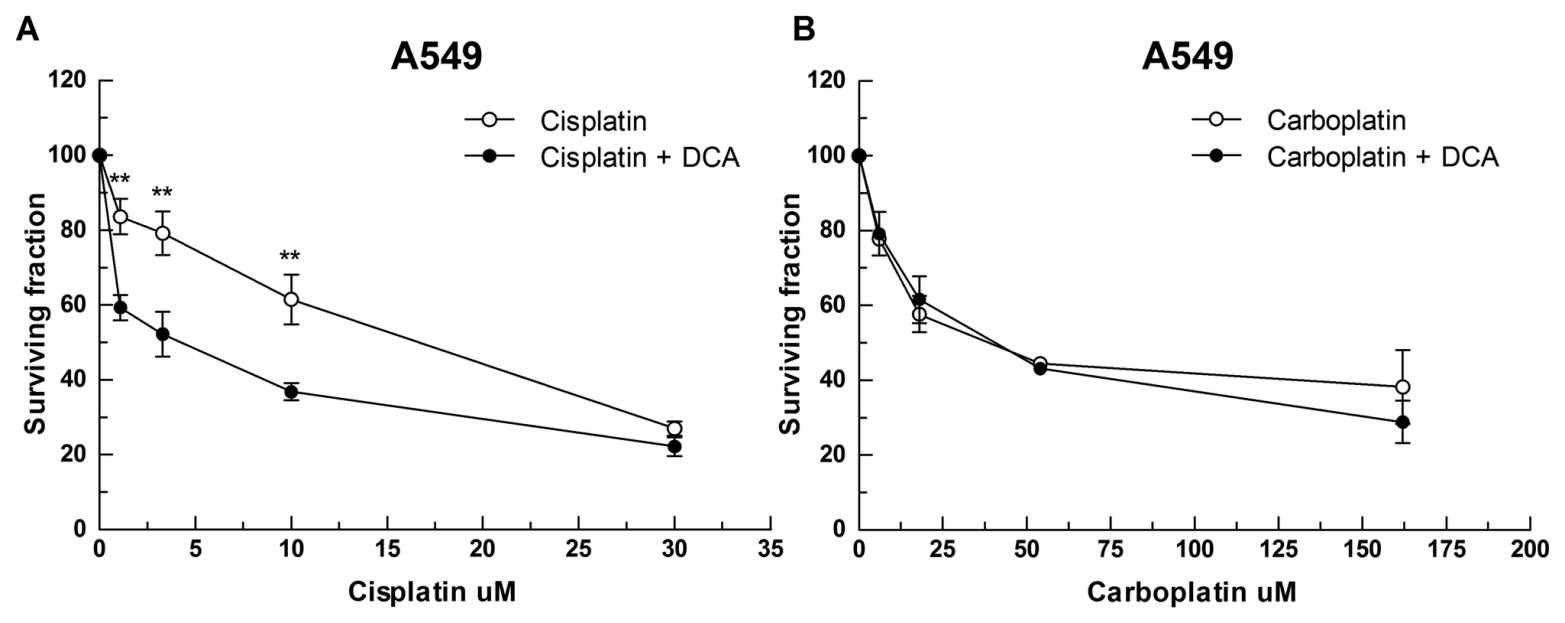
Metabolic state of the cell determines the contribution of the mitochondrial-ROS component to the cytotoxic effect of cisplatin. Survival of A549 cells after 72 h exposure to a dose range of either (**E**) cisplatin or (**F**) carboplatin with or without exposure to 1 mM of DCA. Data represent mean of n=3 independent experiments +/- SD; **p<0.005.

Our results indicate that cisplatin but not carboplatin exposure induces mitochondrial impairment and subsequent promotion of cell death. Based on this model, we predicted that an elevated mitochondrial metabolic activity should enhance cisplatin cytotoxicity in cancer cells as, in this scenario, the damage to mitochondria would lead to a status of metabolic stress. To address this possibility, we exploited the abnormal metabolism of cancer cells. Cancer cells produce energy mainly through glycolysis, even in the presence of oxygen (Warburg effect) [43]. However, aerobic glycolysis can be reverted by promoting mitochondrial glucose oxidation. To validate our prediction we used the pyruvate dehydrogenase kinase inhibitor dichloroacetate (DCA) to switch the metabolism of cancer cells from glycolysis to glucose oxidation [44]. Thus, addition of DCA should sensitize cancer cells to cisplatin, but not to carboplatin. We co-treated A549 cells with cisplatin or carboplatin and a non-toxic dose of DCA (1 mM). It should be noted that exposure to a 0.5 mM dose of DCA has been reported as sufficient to promote glucose oxidation in A549 cells [44] and accordingly, we observed a significant reduction in lactate production following DCA exposure in A549 cells (Figure S9). Exposure to DCA sensitized A549 cells to cisplatin (Figure 6A), but not to carboplatin (Figure 6B). These results support our model and suggest that mitochondrial metabolic activity influences the response of cancer cells to cisplatin-induced toxic effects on mitochondria.

## Discussion

**Figure 7:**
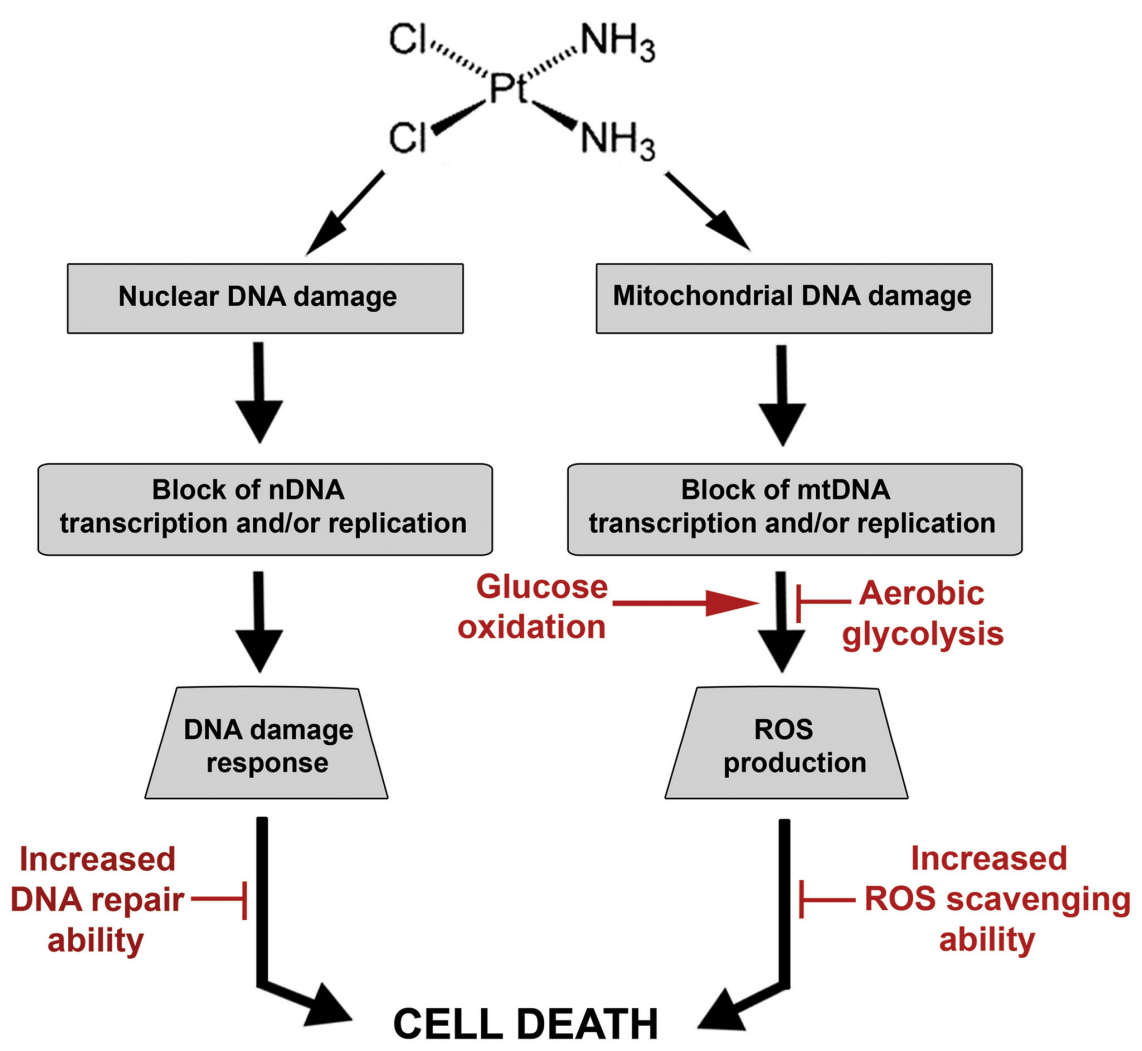
Model for major components of cisplatin-induced cytotoxicity. Cellular exposure to cisplatin causes direct damage to mtDNA resulting in a reduction of mitochondrial protein synthesis, impairment of electron transport chain function, and subsequently, increases in intracellular ROS levels. ROS ultimately promotes cell death, resulting in a significant enhancement of the cytotoxic effect exerted by cisplatin through the generation of nDNA damage. Mitochondrial dysfunction, increased ability to scavenge mitochondrial ROS and glycolytic metabolism reduce cellular sensitivity to the mitochondrial-ROS mediated component of cisplatin cytotoxicity. Reduction in cellular sensitivity to cisplatin can also be achieved by increased DNA repair capacity. Additional, minor components not illustrated in this model may also affect cisplatin cytotoxicity.

In the present study we demonstrate that cisplatin exposure induces a mitochondria-dependent ROS response that significantly contributes to cell killing by enhancing the cytotoxic effect exerted through the formation of nDNA damage. Cisplatin-induced ROS generation occurs as consequence of its direct effect on mtDNA, resulting in the impairment of electron transport chain protein synthesis. We demonstrate that mitochondrial redox status, DNA integrity and bioenergetic functionality are key modulators of the cellular response to cisplatin-induced mitochondrial impairment and may be factors determining resistance to its cytotoxic effect. Therefore, our findings reveal that cisplatin-induced cytotoxicity is mediated by at least two general components whose relative contributions in causing cell death may depend on cell proliferation, redox status and metabolic activity (Figure 7).

 Mitochondrial damage has been observed in several models of cisplatin induced nephrotoxicity and neurotoxicity [16,45–48] and targeted delivery of antioxidants to mitochondria reduces the onset of cisplatin-induced renal cell damage [49]. These observations suggest that mitochondrial oxidative damage constitutes a component for cisplatin dose-limiting toxicities. Although other groups have reported that cisplatin exposure results in mitochondrial injury and energy imbalance [50,51] in cancer cells, the mechanism of cisplatin-induced mitochondrial impairment and subsequent ROS generation has not been previously characterized. Cisplatin exposure results in caspase activation even in enucleated cancer cells [16], suggesting that cisplatin may also kill cancer cells by a mechanism independent of nDNA damage and mediated by damage to other intracellular organelles or macromolecules. Our findings support these observations and here we propose a model that provides a more comprehensive picture of cisplatin cytotoxicity (Figure 7).

Our model of two major components contributing to cisplatin cytotoxicity would also explain the reduced clinical activity and toxicity of carboplatin compared to cisplatin. We demonstrated that carboplatin is less efficient than cisplatin for impairing mitochondrial protein synthesis or increasing intracellular ROS levels in cancer cells, indicating that carboplatin-induced cell killing may be primarily determined by the nuclear-DNA damage component. To our knowledge this is the first time that such a difference in ability to induce mitochondrial impairment and ROS generation among cisplatin and carboplatin has been demonstrated. In testicular cancer, cisplatin-based combination regimens result in a cure for the majority of the patients, but carboplatin-based regimens are associated with sub-optimal outcomes [52,53]; these observations may be explained by our findings. By providing new insights into the differential activity of cisplatin and carboplatin our studies may inform the design of novel platinum-analogs with reduced toxicities but comparable efficacies to cisplatin. 

 By using both mammalian and yeast cell model systems, we demonstrated that generation of ROS is necessary to promote cell death in response to cisplatin-induced mitochondrial damage. ROS can cause cell death either directly or through activation of intracellular pro-apoptotic pathways [54–56]. The tissue specificity of cisplatin cytotoxicity suggests the involvement of components further downstream from mitochondrial ROS generation that results in cell killing. We speculate that the expression and/or the activity of the protein(s) involved in such downstream pathways may vary among cell types, therefore explaining the tissue-specificity of cisplatin dose-limiting toxicities. Several cytoplasmic enzymes have been shown to be activated by ROS and induce cell death [55–57]. In particular, the AMP-activated protein kinase (AMPK) has recently been shown to promote apoptosis specifically in neurons of the inner ear by a mechanism where impairment in mitochondrial protein synthesis results in ROS generation [58]. Considering that auditory toxicity is a common side effect of cisplatin therapy, the AMPK-mediated pro-apoptotic pathway may act by promoting cell death in response to cisplatin-induced mitochondrial oxidative stress. 

 A major limitation of cisplatin therapeutic effectiveness is the development of resistance. We demonstrated that increased mitochondrial ROS scavenging reduces cellular sensitivity to cisplatin, as approximately a three-fold higher dose of cisplatin is required to kill the same percentage of cells expressing mitochondrial catalase compared to control cells. Accordingly, other groups observed increased expression of ROS scavenging enzymes in cisplatin-resistant cancer cells compared to normal cells [59–61]; thus, our findings may serve as a framework to uncover new mechanisms for cisplatin resistance in cancer cells.

One implication of our study is that cellular metabolism may be a key, manipulable determinant of cisplatin cytotoxicity in cancer cells as we observed that addition of an agent that promotes mitochondrial glucose oxidation, such as DCA, augments the cytotoxic effect of cisplatin. In addition to its well documented effect on metabolism, DCA has been reported to modulate the expression of Kv1.5 and survivin in cancer cells [44]. DCA-induced increase in Kv1.5 expression and reduction in survivin levels result into cancer cell sensitization to apoptosis [44]. However, if the influence of DCA on cisplatin cytotoxicity was exclusively due to such a mechanism, DCA should have exerted a similar effect in both cisplatin and carboplatin-exposed cells. Instead, the specificity observed toward cisplatin supports our conclusions as cisplatin but not carboplatin induces mitochondrial impairment in our cells. Moreover, the effects of DCA on Kv1.5 expression levels are controversial [62]. Therefore, although we cannot exclude that additional mechanisms could contribute to DCA-induced enhancement of cisplatin cytotoxicity, our results suggest that the DCA sensitizes cancer cells to cisplatin at least partially by promoting mitochondrial metabolic activity in cancer cells. Supporting this mechanism, WTρ^°^ yeast cells were only slightly resistant to cisplatin, in contrast to what is observed in mammalian ρ^0^ cells. The difference in the extent to which mitochondrial impairment contributes to cisplatin-induced cell death in yeast and mammalian cells may be explained by the fact that yeast, as facultative anaerobic organism, can rely on glucose fermentation for energy production in the event of drug-induced mitochondrial impairment. A technical limitation of our study is the use of cells cultured in glucose-containing media to analyze cisplatin-induced mitochondrial toxicity. Although routinely used for similar studies, immortalized cell lines grown in these conditions derive their energy mainly from glycolysis rather than mitochondrial oxidation, independently of the presence of fully functional mitochondria (Crabtee effect) [63]. Such a metabolic adaptation results in lower susceptibility toward the effects of mitochondrial toxicants on cell viability and growth [64]; therefore, the contribution of cisplatin-induced mitochondrial impairment to its cytotoxic effect may have been underestimated in our study as well as in those performed by other groups. 

Our model has important clinical implications as cancer cell metabolism differs greatly from that of normal cells. It predicts that while the toxic effect exerted by cisplatin in normal, post-mitotic cells may be caused by a combination of mitochondrial impairment and nDNA transcription blockage, the cytotoxic effect of cisplatin on actively replicating, glycolytic cancer cells is likely predominantly due to the nDNA damage component. Therefore, promoting glucose oxidation should significantly increase the cytotoxic effect of cisplatin in cancer cells with minimal effects on normal cells. Moreover, the Warburg effect is often sustained by oncogene-driven pathways that are altered in cancer cells and that may constitute ideal “druggable’ targets for the development of sensitizers for cisplatin treatment [65]. 

In conclusion, our study provides new insight for understanding the high degree of effectiveness of cisplatin compared to other DNA-damaging anticancer agents as well as the basis for cisplatin dose-limiting toxicities.

## Supporting Information

Figure S1 **DU145ρ^°^ cells lack mitochondrial DNA and do not express mtDNA encoded proteins.** The ρ^0^ status of DU145ρ^°^ cells was confirmed by (**A**) PCR amplification of a 460 bp mtDNA fragment as described in Materials and Methods S1. PCR amplification of the nDNA encoded GADPH gene was used as control. (**B**) Western Blot analysis of mitochondrial-encoded cytochrome c oxidase subunit 1 (MT-CO1) and succinate dehydrogenase complex subunit A (SDHA) expression in DU145 and DU145ρ^°^ cells.
(TIF)

Figure S2 **Mitochondrial ROS levels in non-apoptotic cancer cells following exposure to cisplatin.** A549 cells were exposed to cisplatin at an IC50 dose (12 µM) for 24 h; then, cells were co-stained with Annexin V and MitoSox and analyzed by flow cytometry. (**A**-**C**) Annexin V-negative subpopulation (about 15-20%) was gated in each sample in order to exclude the apoptotic fraction of cells from the analysis. (**D**-**F**) Mitochondrial ROS levels in the gated Annexin V-negative sub-population.
(TIF)

Figure S3 **Overexpression of mitochondrial-targeted catalase in mCat cells.** Expression of catalase in mitochondria was evaluated 48 h following transfection in mCat cells by (**A**) Immunofluorescence as described in Materials and Methods S1 and (**B**) Western Blot. For Western Blot analysis catalase antibody was used at a 1:5000 dilution. β-actin expression (1:1000, Sigma-Aldrich) was used as loading control. (**C**) **mCat cells have lower endogenous ROS levels compared to Ctrl cells**. Ctrl and mCat cells were incubated with H_2_DCFDA (10 µM) for 30 minutes and ROS levels determined as described in Materials and Methods. Statistical analysis was performed with Student t-test ** = p<0.005.
(TIF)

Figure S4 **DU145ρ^°^ display lower endogenous ROS levels compared to DU145 cells.** DU145 and DU145ρ^°^ cells were incubated with H_2_DCFDA (10 µM) for 30 minutes and ROS levels determined as described in Materials and Methods. Bars represent the mean of three independent experiments, each performed in triplicate. Statistical analysis was performed with Student t-test
* = p<0.05.
(TIF)

Figure S5 **DU145 parental and DU145ρ^°^ cells are equally susceptible to staurosporine-induced apoptosis.** DU145 and DU145ρ^°^ cells were exposed to a dose range of the apoptosis inducing agent staurosporine and survival was analyzed by crystal violet staining after 48 h. Bars represent the mean of three independent experiments, each performed in triplicate.
(TIF)

Figure S6 **Chloramphenicol exposure induces an increase in mitochondrial ROS levels after 16-24 h of exposure.** A549 cells were continuatively exposed to either cisplatin (12 µM) or chloramphenicol (100 µg/mL) and mitochondrial ROS levels were measured at the indicated time points as described in Materials and Methods. Data are presented as fold increase over no treatment. Bars represent the mean of three independent experiments, each performed in triplicate.
(TIF)

Figure S7 **Exposure to cisplatin and carboplatin did not significantly reduce mRNA levels of ETC protein encoded by nDNA.** A549 cells were exposed to cisplatin and carboplatin at an IC50 dose (12 µM and 50 µM, respectively) and SDHA mRNA levels were analyzed by qRT-PCR as described in Materials and Methods. Bars represent mean of n=5 experiments +/- SE. Data are presented as fold change compared to control (no treatment, black dotted line).
(TIF)

Figure S8 **Co-treatment with the mitochondrial ROS scavenger Mitotempo reduces cisplatin-induced apoptosis in cancer cells.** A549 cells were exposed to cisplatin (12 µM) and Mitotempo (10 µM) either alone or in combination for 48 h and percentage of cell apoptosis was determined by staining with Annexin V and subsequent flow cytometry analysis. Apoptotic cells in treated vs. non treated cells were compared by one-way ANOVA (p<0.05; Bonferroni post-test for multiple comparison: *p<0.05, **p<0.01). Bars represent the mean of three independent experiments, each performed in triplicate.
(TIF)

Figure S9 **Treatment with DCA reduces lactate production in cancer cells.** A549 cells were treated with DCA (1 mM) for 48 h and lactate concentration was determined in cellular media as described in Materials and Methods S1. Bars represent the mean of three independent experiments, each performed in triplicate. The statistical analysis was performed with Student t-test; *p<0.05.
(TIF)

Materials and Methods S1 **Supplementary Materials and Methods.**
(DOC)
