# Supplementary figures and images for: Cisplatin Induces a Mitochondrial-ROS Response That Contributes to Cytotoxicity Depending on Mitochondrial Redox Status and Bioenergetic Functions
Source: PLoS One. 2013 Nov 19;8(11):e81162. doi: 10.1371/journal.pone.0081162 (PMC3834214; doi:10.1371/journal.pone.0081162)

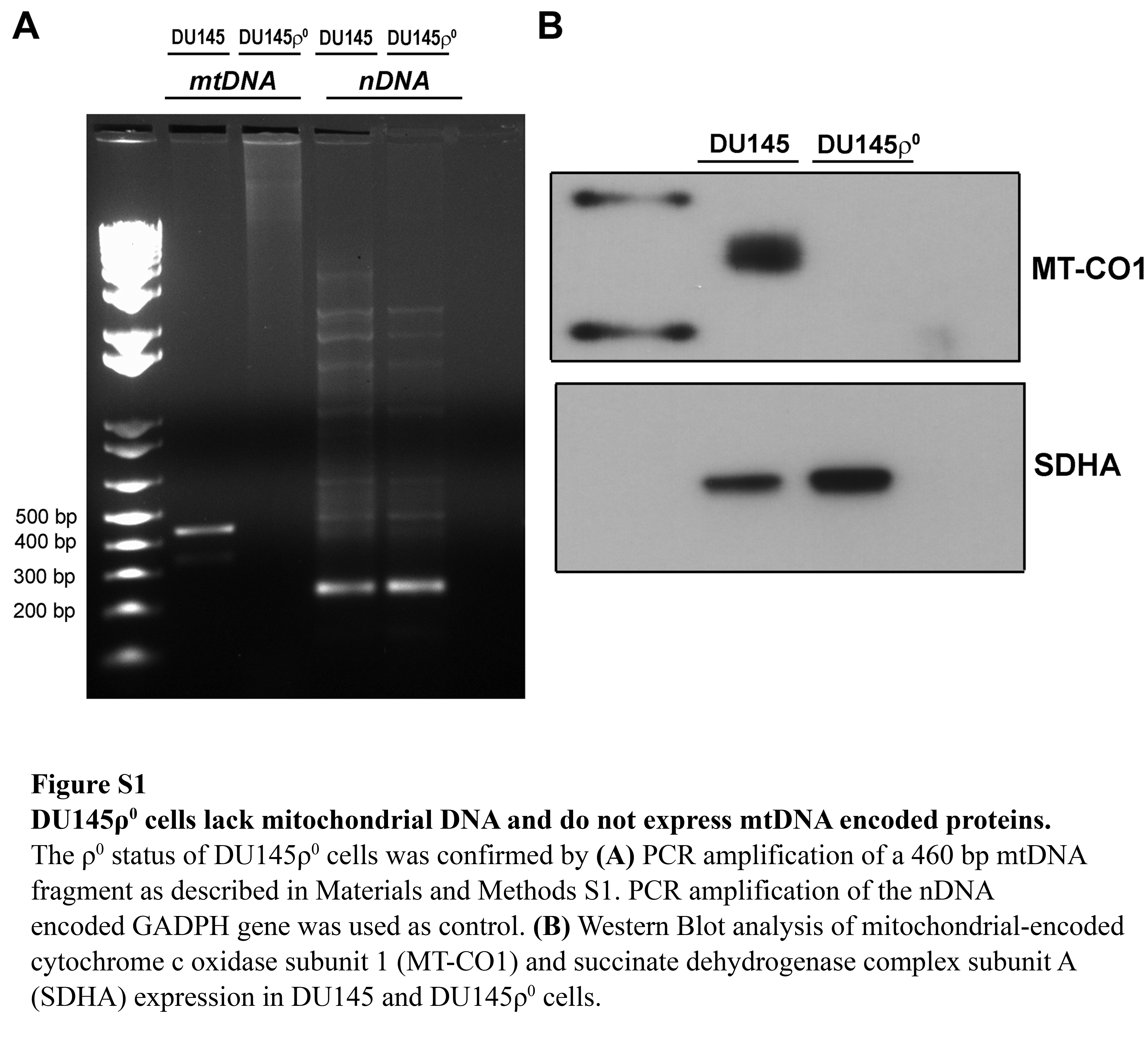

Supplement: Figure S1 — DU145ρ° cells lack mitochondrial DNA and do not express mtDNA encoded proteins. The ρ0 status of DU145ρ° cells was confirmed by (A) PCR amplification of a 460 bp mtDNA fragment as described in Materials and Methods S1. PCR amplification of the nDNA encoded GADPH gene was used as control. (B) Western Blot analysis of mitochondrial-encoded cytochrome c oxidase subunit 1 (MT-CO1) and succinate dehydrogenase complex subunit A (SDHA) expression in DU145 and DU145ρ° cells. (TIF) [file pone.0081162.s001.tif]

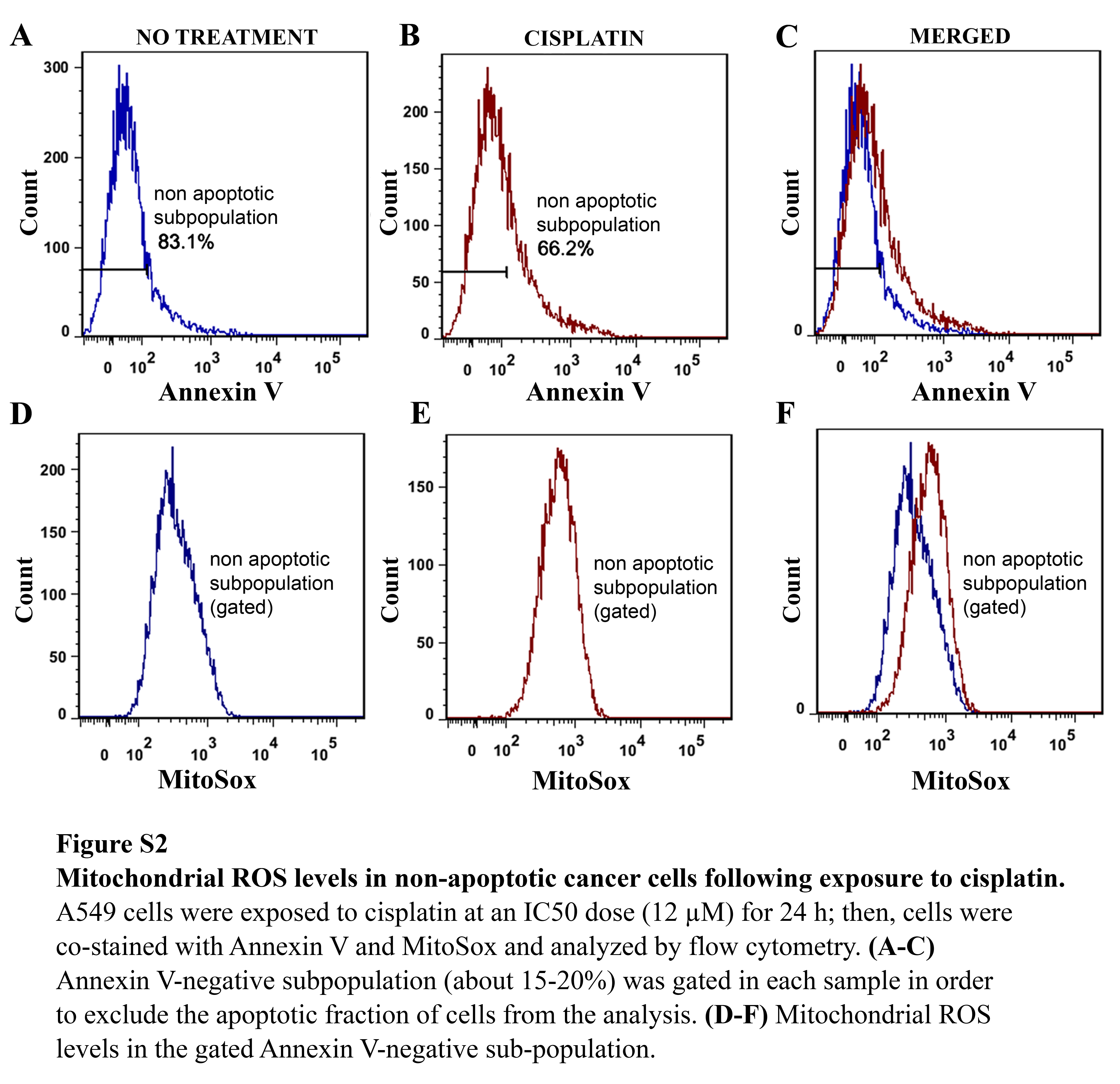

Supplement: Figure S2 — Mitochondrial ROS levels in non-apoptotic cancer cells following exposure to cisplatin. A549 cells were exposed to cisplatin at an IC50 dose (12 µM) for 24 h; then, cells were co-stained with Annexin V and MitoSox and analyzed by flow cytometry. (A-C) Annexin V-negative subpopulation (about 15-20%) was gated in each sample in order to exclude the apoptotic fraction of cells from the analysis. (D-F) Mitochondrial ROS levels in the gated Annexin V-negative sub-population. (TIF) [file pone.0081162.s002.tif]

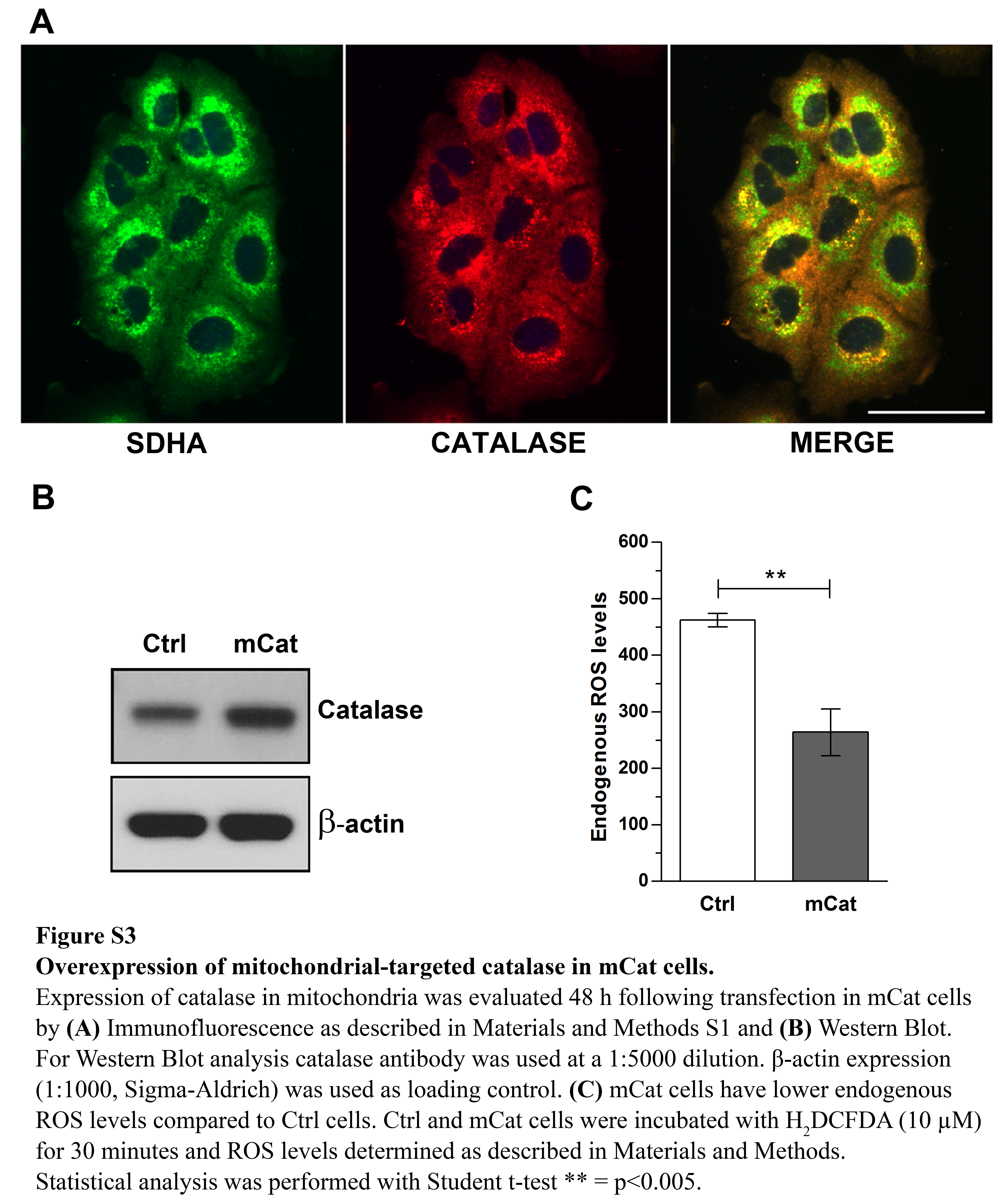

Supplement: Figure S3 — Overexpression of mitochondrial-targeted catalase in mCat cells. Expression of catalase in mitochondria was evaluated 48 h following transfection in mCat cells by (A) Immunofluorescence as described in Materials and Methods S1 and (B) Western Blot. For Western Blot analysis catalase antibody was used at a 1:5000 dilution. β-actin expression (1:1000, Sigma-Aldrich) was used as loading control. (C) mCat cells have lower endogenous ROS levels compared to Ctrl cells. Ctrl and mCat cells were incubated with H2DCFDA (10 µM) for 30 minutes and ROS levels determined as described in Materials and Methods. Statistical analysis was performed with Student t-test ** = p<0.005. (TIF) [file pone.0081162.s003.tif]

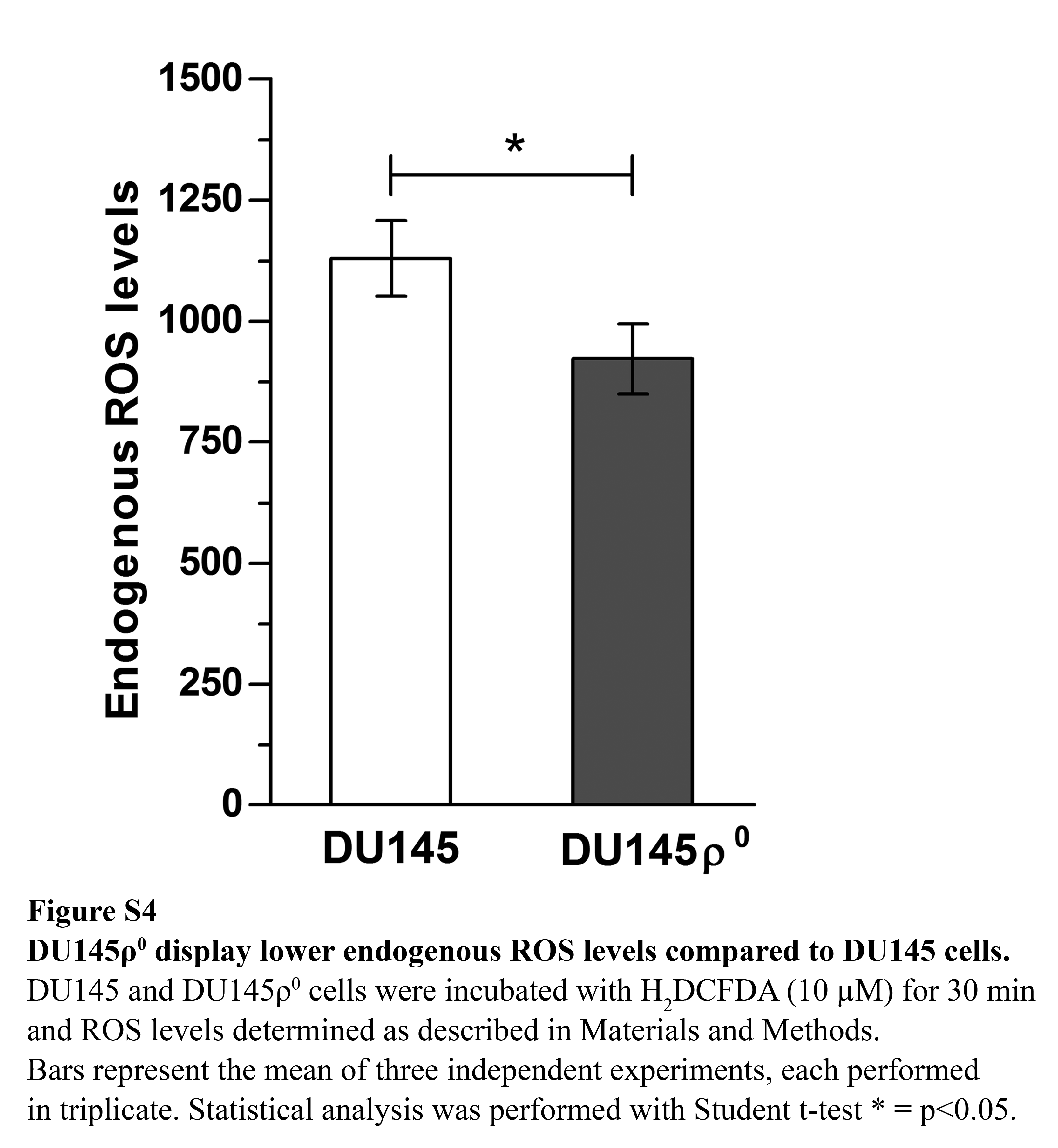

Supplement: Figure S4 — DU145ρ° display lower endogenous ROS levels compared to DU145 cells. DU145 and DU145ρ° cells were incubated with H2DCFDA (10 µM) for 30 minutes and ROS levels determined as described in Materials and Methods. Bars represent the mean of three independent experiments, each performed in triplicate. Statistical analysis was performed with Student t-test * = p<0.05. (TIF) [file pone.0081162.s004.tif]

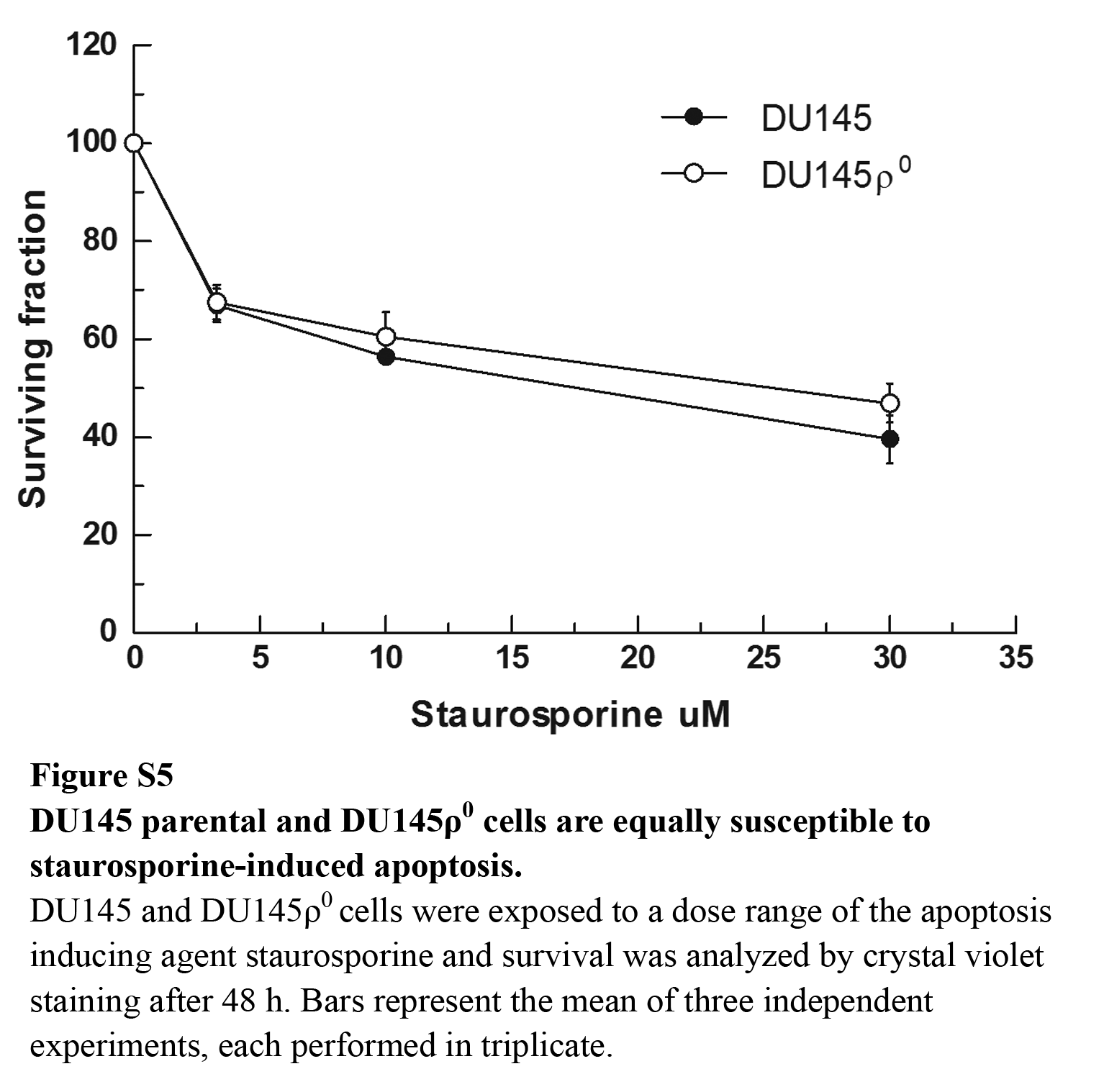

Supplement: Figure S5 — DU145 parental and DU145ρ° cells are equally susceptible to staurosporine-induced apoptosis. DU145 and DU145ρ° cells were exposed to a dose range of the apoptosis inducing agent staurosporine and survival was analyzed by crystal violet staining after 48 h. Bars represent the mean of three independent experiments, each performed in triplicate. (TIF) [file pone.0081162.s005.tif]

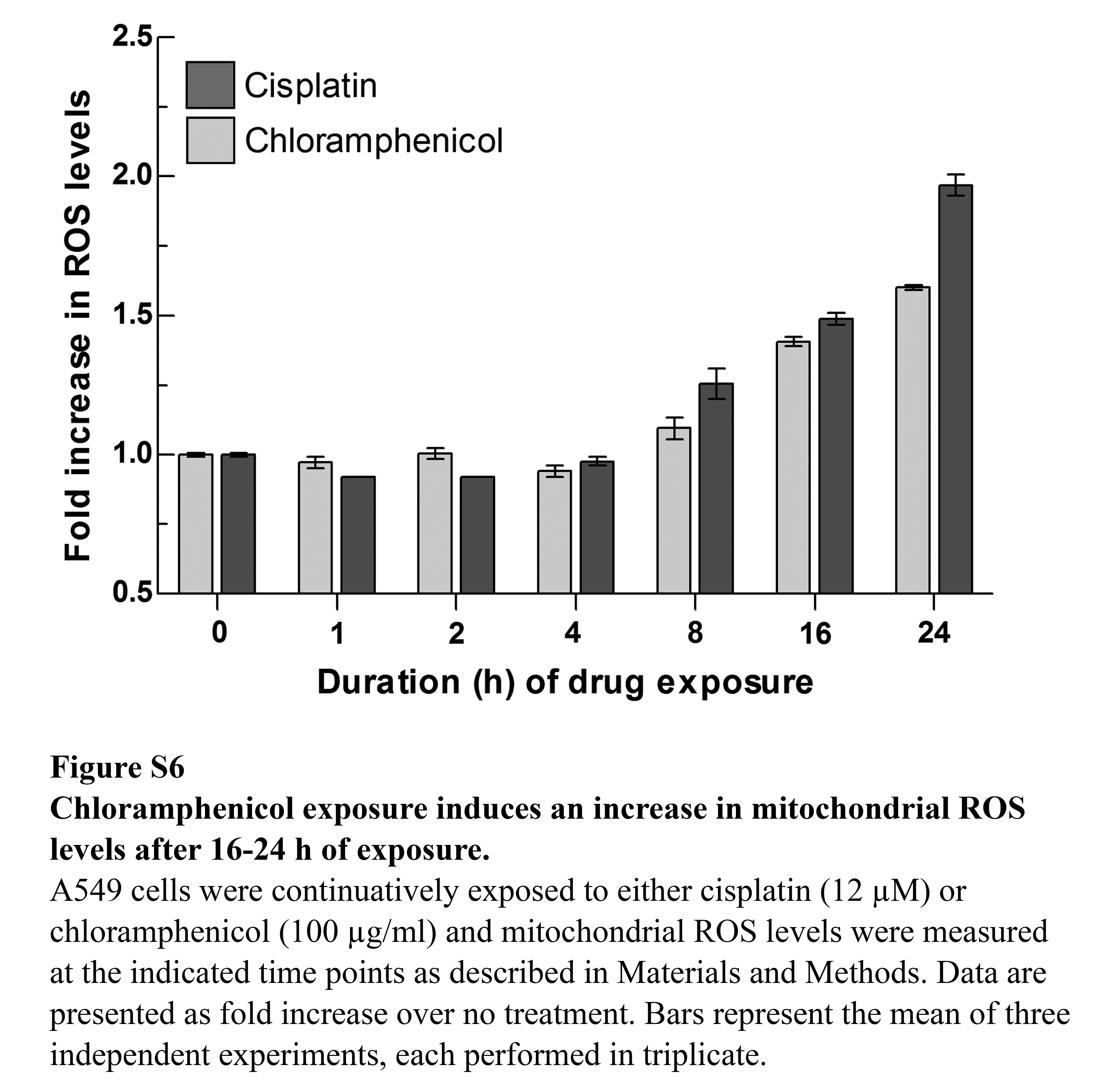

Supplement: Figure S6 — Chloramphenicol exposure induces an increase in mitochondrial ROS levels after 16-24 h of exposure. A549 cells were continuatively exposed to either cisplatin (12 µM) or chloramphenicol (100 µg/mL) and mitochondrial ROS levels were measured at the indicated time points as described in Materials and Methods. Data are presented as fold increase over no treatment. Bars represent the mean of three independent experiments, each performed in triplicate. (TIF) [file pone.0081162.s006.tif]

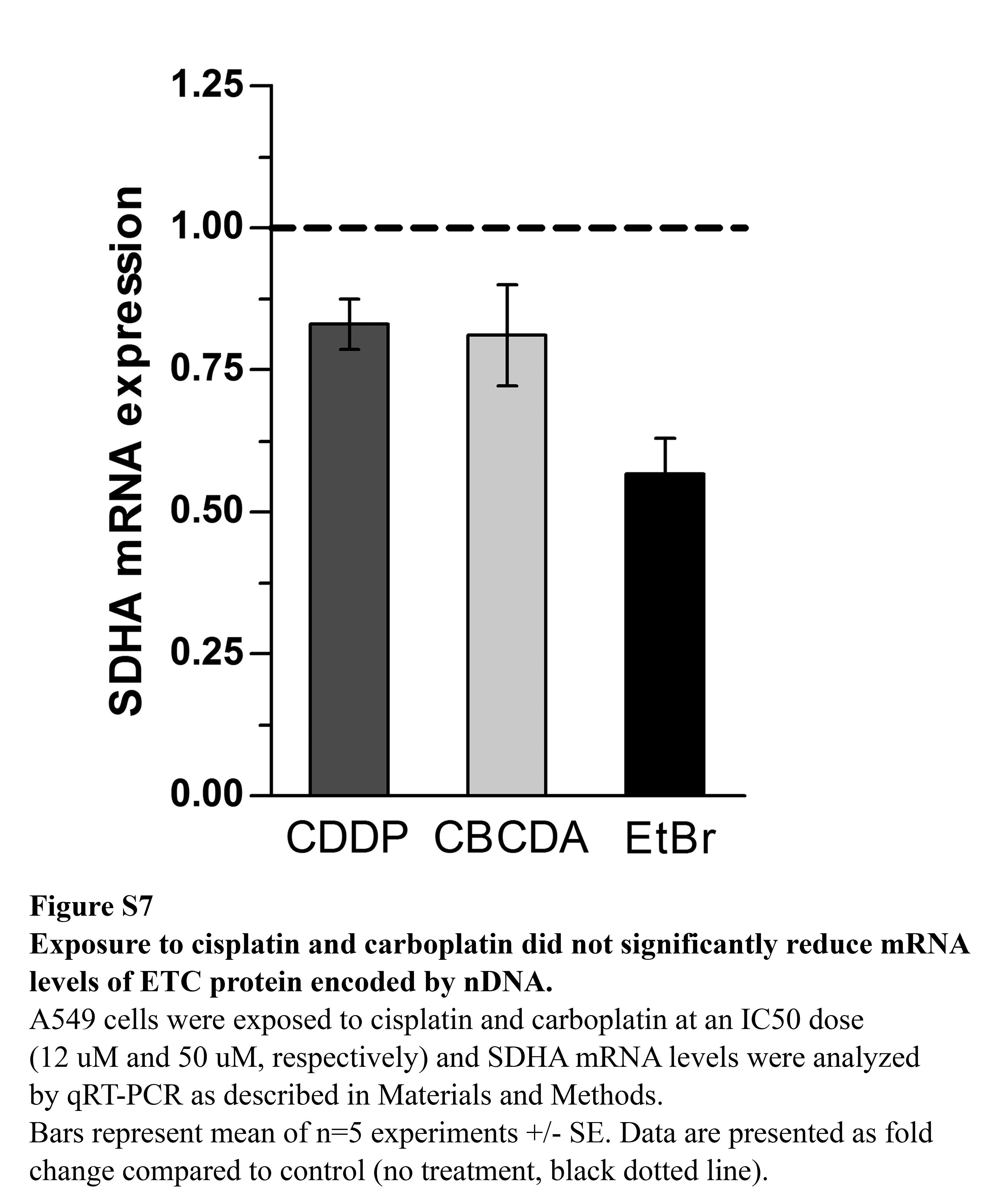

Supplement: Figure S7 — Exposure to cisplatin and carboplatin did not significantly reduce mRNA levels of ETC protein encoded by nDNA. A549 cells were exposed to cisplatin and carboplatin at an IC50 dose (12 µM and 50 µM, respectively) and SDHA mRNA levels were analyzed by qRT-PCR as described in Materials and Methods. Bars represent mean of n=5 experiments +/- SE. Data are presented as fold change compared to control (no treatment, black dotted line). (TIF) [file pone.0081162.s007.tif]

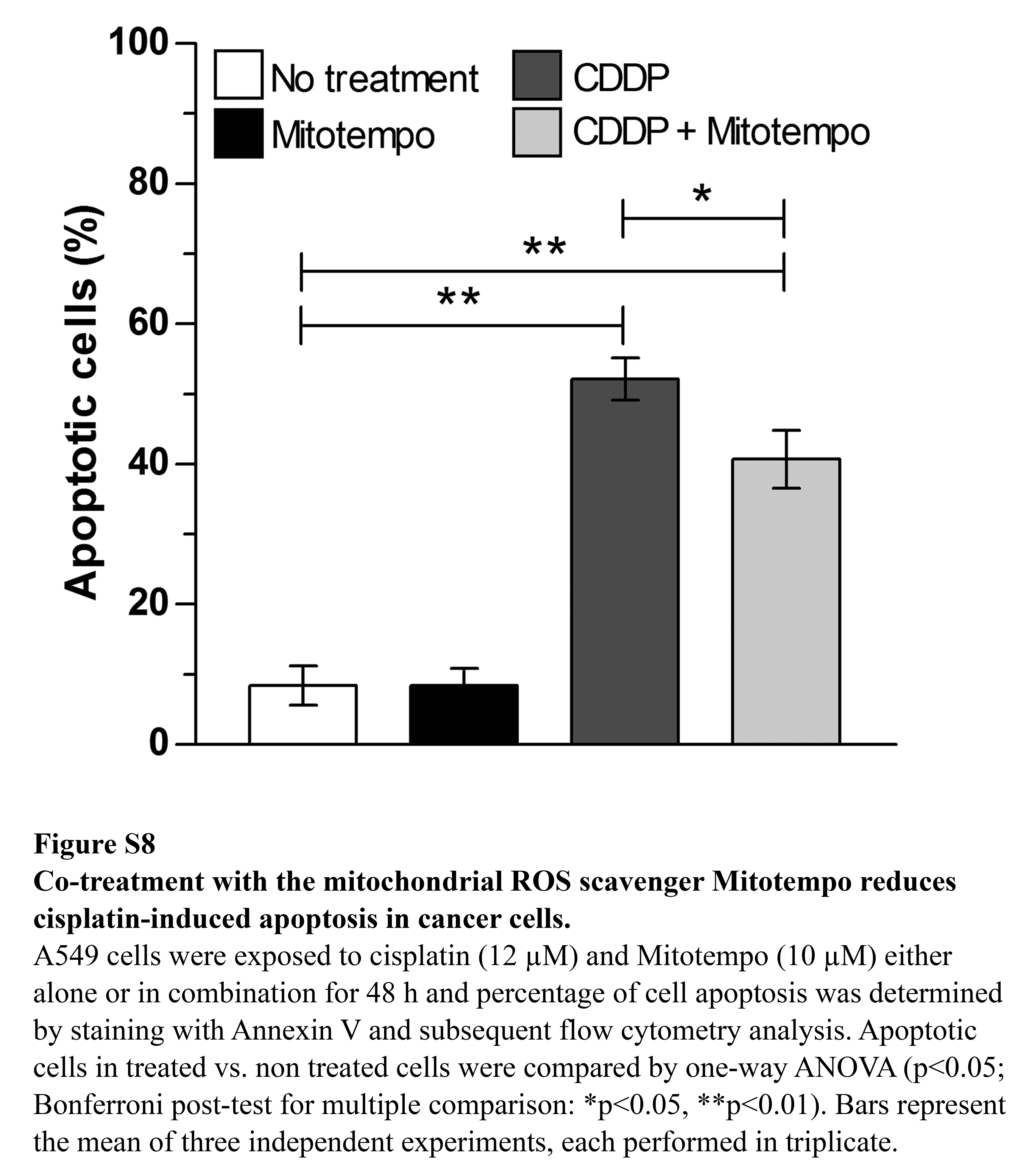

Supplement: Figure S8 — Co-treatment with the mitochondrial ROS scavenger Mitotempo reduces cisplatin-induced apoptosis in cancer cells. A549 cells were exposed to cisplatin (12 µM) and Mitotempo (10 µM) either alone or in combination for 48 h and percentage of cell apoptosis was determined by staining with Annexin V and subsequent flow cytometry analysis. Apoptotic cells in treated vs. non treated cells were compared by one-way ANOVA (p<0.05; Bonferroni post-test for multiple comparison: *p<0.05, **p<0.01). Bars represent the mean of three independent experiments, each performed in triplicate. (TIF) [file pone.0081162.s008.tif]

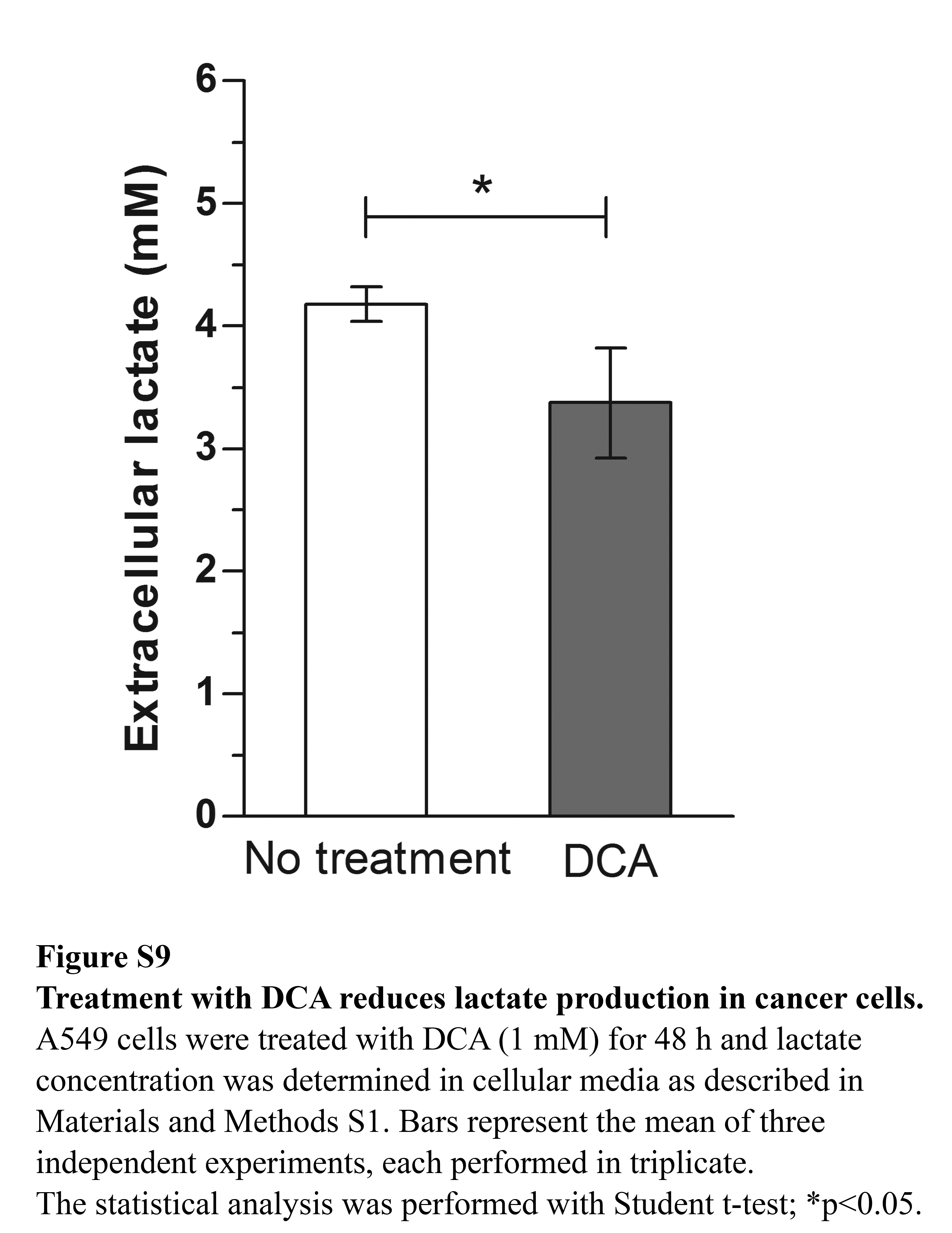

Supplement: Figure S9 — Treatment with DCA reduces lactate production in cancer cells. A549 cells were treated with DCA (1 mM) for 48 h and lactate concentration was determined in cellular media as described in Materials and Methods S1. Bars represent the mean of three independent experiments, each performed in triplicate. The statistical analysis was performed with Student t-test; *p<0.05. (TIF) [file pone.0081162.s009.tif]
