# Supplementary material for: Cisplatin Induces a Mitochondrial-ROS Response That Contributes to Cytotoxicity Depending on Mitochondrial Redox Status and Bioenergetic Functions
Source: PLoS One. 2013 Nov 19;8(11):e81162. doi: 10.1371/journal.pone.0081162 (PMC3834214; doi:10.1371/journal.pone.0081162)
Supplement: Materials and Methods S1 — Supplementary Materials and Methods. (DOC) [file pone.0081162.s010.doc]

**Supplementary Materials and Methods**

**Rho^0^ status verification**

Total DNA was isolated from approximately 5x10^5^ cells using MasterPure Complete DNA & RNA Purification Kit (Epicentre Biotechnologies) according to the manufacturer`s protocol. A 461 bp fragment of mtDNA was generated using 1 µg of DNA and the following PCR conditions: 30 cycles of 94°C for 30 seconds, 52°C for 30 seconds and 72°C for 60 seconds. Primers used for mtDNA fragment amplification were the following: forward primer 5’TGTACGAAAGGACAAGAGAA 3’ and reverse primer 5’TAGAAGAGCGATGGTGAGAG 3’. PCR amplification of the nDNA encoded GADPH gene was used as control. Primers used for GADPH amplification were the following: forward primer 5`GGAAGGTGAAGGTCGGAGT 3`and reverse primer 5`GAAGATGGTGATGGGATTTC 3`.

**Immunofluorescence**

To confirm the expression of catalase in mitochondria, approximately 5x10^4^ mCat cells were plated on a glass coverslip 48 h following transfection with a plasmid containing mitochondrial-targeted catalase. Cells were fixed with 4% paraformaldehyde (10 minutes) and permeabilized with 0.2% Triton X 100 in PBS (5 minutes). Cells were then incubated with blocking solution (10% FBS in PBS) for 1 h. Catalase expression was evaluated using anti-catalase rabbit antibody (1:500, 1 h exposure; Calbiochem). SDHA expression (1:500, overnight exposure at 4°C; MitoSciences) was used as mitochondrial marker. The secondary antibodies were Alexa Fluor® 568 goat anti-rabbit (1:1000, 1 h; Invitrogen) and Alexa Fluor® 488 goat anti-mouse (1:1000, 1 h; Invitrogen). DAPI was used as nuclear staining. Pictures were captured using Olympus IX81 inverted microscope and SlideBook^TM^ software.

**ROS measurement in MEFs cells**

WT and TFAM^+/-^ MEFs were plated on a 12 well plate in triplicate the day before the experiment. Media was then replaced with fresh media containing cisplatin at an IC50 dose (10 μM). Following 24 h of exposure ROS levels were measured by using Amplex Red (Invitrogen) in the presence of horseradish-peroxidase according to the manufacturer`s protocol. Exogenous catalase was used as control. Fluorescence was measured using a SpectraMax M5 Plate Reader. Crystal violet staining was used to normalize ROS levels to cell number. Catalase-abolished signal from three independent experiments was used to calculate the ROS levels in these cells.

**Determination of cell apoptosis by Annexin V staining**

Cells were plated on a 6 well plate the day before the experiment. Media was then replaced with fresh media containing cisplatin (12 μM) and Mitotempo (10 μM; Enzo Life Science) alone or in combination. Following 48 h of exposure cells were collected, washed 2 times with PBS and apoptosis was measured with Alexa Fluor 488/Annexin V Dead Cell Apoptosis Kit (Life Technologies) according to the manufacturer`s protocol. Samples were analyzed within 30 min with a BD LSR II flow cytometer (BD Biosciences). Data represent the mean of three independent experiments, each performed in triplicate.

**Determination of extracellular lactate production**

Cells were plated on a 24 well plate the day before the experiment. Media was then replaced with fresh media (300 μL) containing DCA (1 mM). Following 48 h of exposure media was collected and deproteinized by centrifugation with a 10kDa MWCO spin filter. Lactate concentration was then measured by using the Lactate Assay Kit (Sigma-Aldrich) according to manufacturer`s protocol. Crystal violet staining was used to normalize lactate concentration to cell number.
